# A Universal Next-Generation Sequencing Protocol To Generate Noninfectious Barcoded cDNA Libraries from High-Containment RNA Viruses

**DOI:** 10.1128/mSystems.00039-15

**Published:** 2016-06-07

**Authors:** Lindsey A. Moser, Lisbeth Ramirez-Carvajal, Vinita Puri, Steven J. Pauszek, Krystal Matthews, Kari A. Dilley, Clancy Mullan, Jennifer McGraw, Michael Khayat, Karen Beeri, Anthony Yee, Vivien Dugan, Mark T. Heise, Matthew B. Frieman, Luis L. Rodriguez, Kristen A. Bernard, David E. Wentworth, Timothy B. Stockwell, Reed S. Shabman

**Affiliations:** a Department of Pathobiological Sciences, University of Wisconsin—Madison, Madison, Wisconsin, USA; b Plum Island Animal Disease Center, Agricultural Research Service, U.S. Department of Agriculture, Greenport, New York, USA; c Virology Group, J. Craig Venter Institute, Rockville, Maryland, USA; d Department of Microbiology and Immunology, University of Maryland at Baltimore, Baltimore, Maryland, USA; e Department of Genetics, University of North Carolina at Chapel Hill, Chapel Hill, North Carolina, USA; f Plum Island Animal Disease Center-Oak Ridge Institute for Science and Education (ORISE) Research Participation Program, Oak Ridge, Tennessee, USA; g Sequencing Group, J. Craig Venter Institute, La Jolla, California, USA; Dalhousie University

**Keywords:** next-generation sequencing, West Nile virus, alphavirus, coronavirus, flavivirus, foot-and-mouth disease virus, genomics, picornavirus, rhinovirus

## Abstract

Several biosafety level 3 and/or 4 (BSL-3/4) pathogens are high-consequence, single-stranded RNA viruses, and their genomes, when introduced into permissive cells, are infectious. Moreover, many of these viruses are select agents (SAs), and their genomes are also considered SAs. For this reason, cDNAs and/or their derivatives must be tested to ensure the absence of infectious virus and/or viral RNA before transfer out of the BSL-3/4 and/or SA laboratory. This tremendously limits the capacity to conduct viral genomic research, particularly the application of next-generation sequencing (NGS). Here, we present a sequence-independent method to rapidly amplify viral genomic RNA while simultaneously abolishing both viral and genomic RNA infectivity across multiple single-stranded positive-sense RNA (ssRNA+) virus families. The process generates barcoded DNA amplicons that range in length from 300 to 1,000 bp, which cannot be used to rescue a virus and are stable to transport at room temperature. Our barcoding approach allows for up to 288 barcoded samples to be pooled into a single library and run across various NGS platforms without potential reconstitution of the viral genome. Our data demonstrate that this approach provides full-length genomic sequence information not only from high-titer virion preparations but it can also recover specific viral sequence from samples with limited starting material in the background of cellular RNA, and it can be used to identify pathogens from unknown samples. In summary, we describe a rapid, universal standard operating procedure that generates high-quality NGS libraries free of infectious virus and infectious viral RNA.

**IMPORTANCE** This report establishes and validates a standard operating procedure (SOP) for select agents (SAs) and other biosafety level 3 and/or 4 (BSL-3/4) RNA viruses to rapidly generate noninfectious, barcoded cDNA amenable for next-generation sequencing (NGS). This eliminates the burden of testing all processed samples derived from high-consequence pathogens prior to transfer from high-containment laboratories to lower-containment facilities for sequencing. Our established protocol can be scaled up for high-throughput sequencing of hundreds of samples simultaneously, which can dramatically reduce the cost and effort required for NGS library construction. NGS data from this SOP can provide complete genome coverage from viral stocks and can also detect virus-specific reads from limited starting material. Our data suggest that the procedure can be implemented and easily validated by institutional biosafety committees across research laboratories.

## Abstract (precis)

This report establishes and validates a standard operating procedure (SOP) for select agents (SAs) and other biosafety level 3 and/or 4 (BSL-3/4) RNA viruses to rapidly generate noninfectious, barcoded cDNA amenable for next-generation sequencing (NGS). This eliminates the burden of testing all processed samples derived from high-consequence pathogens prior to transfer from high-containment laboratories to lower-containment facilities for sequencing. Our established protocol can be scaled up for high-throughput sequencing of hundreds of samples simultaneously, which can dramatically reduce the cost and effort required for NGS library construction. NGS data from this SOP can provide complete genome coverage from viral stocks and can also detect virus-specific reads from limited starting material. Our data suggest that the procedure can be implemented and easily validated by institutional biosafety committees across research laboratories.

## INTRODUCTION

Single-stranded positive-sense RNA (ssRNA+) viruses constitute the largest group of viral agents (1). Even when not encapsidated by viral structural proteins, positive-sense RNA genomes are sufficient to initiate translation and viral replication upon introduction into permissive cells. The infectious nature of ssRNA+ viral genomes has made them amenable to reverse genetic manipulation for more than 30 years (2, 3). However, this property is a contributing factor in classifying many ssRNA+ viruses as select agents (SAs) under the Federal Select Agent Program (4). Moreover, many ssRNA+ viruses are high-priority biosafety level 3 and/or 4 (BSL-3/4) pathogens, with a subset listed as potential bioterrorism agents by the U.S. Department of Health and Human Services (5, 6). An additional complication is that full-length cDNAs from ssRNA+ viruses that are SAs can be used to create RNA and rescue/recover the pathogen. Thus, in some instances, large cDNAs, double-stranded DNAs (dsDNAs), or clones containing at least two-thirds of the genome may also be regulated as SAs.

Positive-sense RNA viruses span multiple virus families, and the infectious nature of these genomic RNAs coupled with SA/biosafety/biosecurity concerns inhibit rapid removal from BSL-3/4 containment (7) or transport and handling of RNA samples that are known to contain viral genomes from outbreak settings. This poses a challenge for timely sample processing and sequence analysis, which could significantly hamper responses during outbreak situations. Typically, all products generated from infectious material must be proven to no longer contain infectious viral particles or infectious genomic RNA prior to transfer to a BSL-2 space. Confirming loss of infectivity (LOI) typically occurs via blind infectivity testing, where a subset of the material is placed on a permissive cell line for at least three subsequent passages (8). For this reason, transferring cDNAs or other nucleic acids from a BSL-3/4 laboratory to a BSL-2 laboratory is a very difficult and time-consuming process for many laboratories. Some investigators have worked out procedures that have been approved by their institutional biosafety committee; however, these procedures vary from institution to institution. For example, some BSL-3 facilities work with mosquito-transmitted ssRNA+ viruses including West Nile virus (WNV) and Chikungunya virus (CHIKV) that are not considered SAs. Other BSL-3 facilities contain ssRNA+ viruses such as severe acute respiratory syndrome coronavirus (SARS-CoV) and Venezuelan equine encephalitis virus (VEEV) that are classified as SAs, but these facilities have safety protocols that allow transfer of genomic RNA or cDNA from BSL-3 to BSL-2 directly by scientists. Finally, there are BSL-3 facilities working with SAs such as foot-and-mouth disease virus (FMDV) at the Plum Island Animal Disease Center (PIADC). Here, only trained safety personnel are allowed to transfer cDNA samples from high-containment laboratories to BSL-2 laboratories after inactivation has been carried out under a validated protocol. For each of these scenarios, a robust, universal standard operating procedure (SOP) to eliminate infectious material while rapidly generating next-generation sequencing (NGS) libraries is critically needed.

FMDV illustrates the majority of the SA, biosafety, and biosecurity issues surrounding high-consequence RNA viruses (9). Among the foreign animal disease viruses, FMDV is the most contagious and has historically set standards for biosafety and biosecurity policies and procedures. The required biosafety level to carry out any infectious virus work with FMDV is biosafety level 3 agriculture (BSL-3Ag), and currently, the only facility authorized to work with FMDV in the United States is PIADC (BSL-3Ag safety considerations reviewed in references 10 and 11). The fact that the viral RNA and RNA derived from infected samples can be infectious when transfected or electroporated into susceptible cells results in strict regulation of any nucleic acid derived from FMDV-infected material. Currently, the only approved methodology to remove FMDV nucleic acids from the BSL-3Ag laboratory at PIADC involves harsh alkaline and thermal treatment that potentially has a deleterious effect on putative NGS libraries (12; M. McIntosh, personal communication). In addition, removal of material derived from diverse BSL-3/4 ssRNA+ pathogens out of containment requires time-consuming procedures (e.g., multiple blind infectivity passages) to rule out the presence of infectious material. This tremendously limits the capacity to conduct genomic research with viral samples, particularly the application of NGS techniques to understand viral pathogenesis, viral ecology, and vaccine development.

For the reasons presented, there is substantial need for a universal SOP to generate cDNA that rapidly and reproducibly inactivates BSL-3/4 viruses that can be easily assessed by institutional biosafety committees (IBC), which speeds the transfer of nucleic acids from high-containment laboratories to BSL-2 laboratories, and enables rapid introduction into a variety of NGS pipelines. Here, we present a robust SOP for generating high-quality, barcoded cDNAs directly from genomic RNA across multiple virus families. Families represented include *Picornaviridae*, *Alphaviridae*, *Flaviviridae*, and *Coronaviridae*, which have genome sizes from ~7 kb to 28 kb. The strategy builds upon established sequence-independent single-primer amplification (SISPA) methods (13-15). Our data prove that barcoded NGS sequencing libraries can be rapidly generated while simultaneously destroying both viral particle and genomic RNA infectivity. Our approach is scalable, highly adaptable, and sensitive. The SOP generates high-quality sequences spanning the entire genome, up to 288 pooled barcoded samples can be examined in a single NGS run, and the products of the SOP work on multiple NGS platforms (e.g., Illumina MiSeq, HiSeq, NextSeq, and Ion Torrent). The SOP works on starting material of purified virus, tissue culture samples, or tissue samples. We are able to detect virus-specific reads in samples where the input is fewer than 10 PFU and can identify viruses present in an unknown sample. Therefore, this application has potential for rapid sequencing of high-titer viral stocks as well as virus discovery and/or forensics. Finally, the nonspecific nature of the amplification makes this SOP adaptable to negative-strand RNA viruses (ssRNA−), double-strand RNA (dsRNA), single-strand DNA (ssDNA), or double-strand DNA (dsDNA) viruses.

## RESULTS

### The SOP rapidly generates noninfectious, barcoded DNA libraries for ssRNA+ viruses.

**FIG 1:**
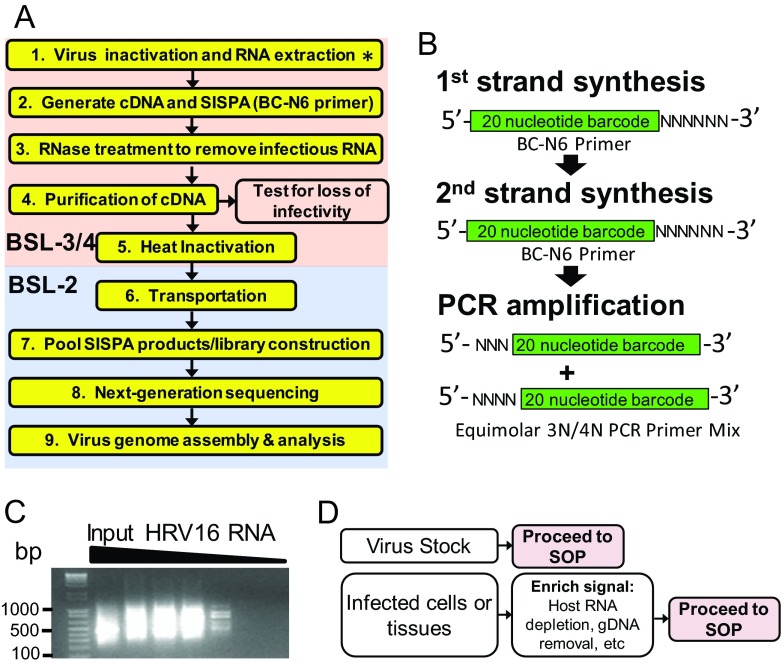
Overview of the proposed standard operating procedure (SOP) for rapid next-generation sequencing library preparation and inactivation of ssRNA+ viruses. (A) Stepwise overview of the SOP. A detailed protocol is provided in Text S1 in the supplemental material. Steps in the pink box denote work performed in a biosafety level 3 and/or 4 (BSL-3/4) laboratory. Steps in the blue box denote work that can be performed in a BSL-2 laboratory. The asterisk in step 1 indicates that for nonselect agent pathogens (e.g., West Nile virus), extracted RNA may be moved to BSL-2 for library construction. Step 1, generating cDNA and SISPA, utilizes a primer with a random hexamer coupled to a unique barcode (BC-N6). SISPA stands for sequence-independent single-primer amplification. (B) The BC-N6 primer is used for both generating single-stranded cDNA from input RNA and generating double-stranded DNA by randomly priming the synthesized cDNA. A PCR step using primers only encoding the barcode sequence with either three or four random nucleotides (3N/4N) at the 5′ end simultaneously amplifies and uniquely identifies (barcodes) a sample. (C) Representative gel image that displays products of the SOP obtained from serial dilutions of genomic human rhinovirus 16 (HRV-16) virion RNA. At high-input RNA amounts, a smear between 200 bp and 1,000 bp is visible. This signal intensity diminishes as the starting material is diluted. (D) Summary of diverse types of starting material which can feed into the SOP. Samples enriched for virus-specific sequence (e.g., virion stocks) can directly proceed to the SOP. For samples that contain a majority of host nucleic acid, the use of upstream procedures to enrich for virus-specific signal (e.g., rRNA depletion or mRNA enhancement) is recommended.

An overview of our approach illustrates nine major steps (Fig. 1A). First, viral RNA is isolated using a commercially available RNeasy kit or Trizol. Second, purified viral RNA is used as a template for sequence-independent cDNA synthesis, and a random library of barcoded PCR products is constructed. Third, DNA products of the SOP are treated with RNase. Fourth, SOP products are purified. At this point, we tested all products of the SOP for loss of viral infectivity and for the absence of infectious full-length viral genomic RNA. This step is not highlighted yellow in Fig. 1 and is not included in the final SOP, since the methods are virus specific. Fifth, immediately prior to transfer, all samples are heated at 72°C for 30 min to inactivate any potential virus contamination that could inadvertently occur during cDNA generation and purification (16). Sixth, all samples are transferred from BSL-3/4 to BSL-2 space. Seventh, products from the SOP are pooled and subjected to NGS library construction. Eighth, libraries are sequenced. Ninth, sequence data are analyzed and computationally assembled in standard office space. A full detailed protocol for the SOP is provided in our supplemental methods (Text S1 in the supplemental material).

10.1128/mSystems.00039-15.5 Text S1  Universal standard operating procedure for generation, barcoding, and amplification of cDNA from genomic RNA from BSL-3/4 viruses. Download Text S1, DOCX file, 0.2 MB. Copyright © 2016 Moser et al. 2016 Moser et al. This content is distributed under the terms of the Creative Commons Attribution 4.0 International license.

Sequence-independent single-primer amplification (SISPA) utilizes a random hexamer primer coupled to a unique barcode (BC-N6 [Fig. 1B]). The BC-N6 oligonucleotide generates single-stranded cDNA from input RNA and double-stranded DNA by randomly priming the synthesized cDNA. Finally, a PCR step with primers encoding only the barcode sequence amplifies and uniquely barcodes a sample. As a result, small PCR products are generated with unique barcodes on each terminus of randomly primed amplicons. A representative gel image displays products of the SOP obtained from serial dilutions of genomic human rhinovirus 16 (HRV-16) virion RNA. At high input RNA amounts, a smear between 200 bp and 1 kb is visible. This signal intensity diminishes as the starting material is diluted (Fig. 1C).

Upstream steps may be required prior to initiating the SOP. When starting from a high-titer virus stock, directly proceeding to the SOP (Fig. 1A) will provide the investigator with sufficient sample purity to obtain viral sequence data that spans the majority of the viral genome. For a heterogeneous sample (e.g., virus-infected cells), a host RNA depletion step, such as rRNA or mRNA removal may be required to enrich for virus-specific sequences before proceeding to the SOP (Fig. 1D).

### The SOP recovers full-length genomic sequence data across multiple ssRNA+ virus families.

**FIG 2:**
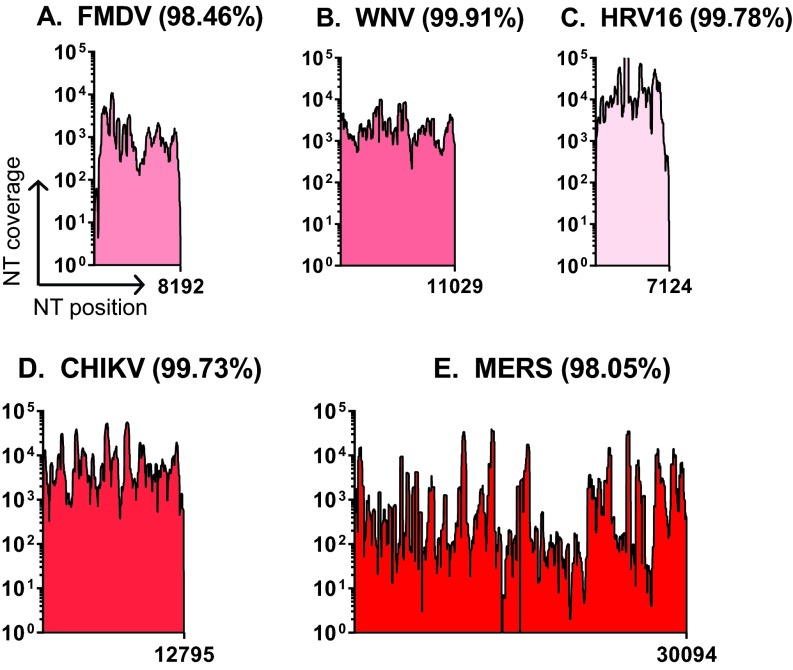
The SOP generates high-quality full-genome sequence data across multiple ssRNA+ virus families. Pooled samples from the SOP were sequenced on the Illumina MiSeq platform. Samples were demultiplexed, the adaptors were trimmed, and low-quality sequencing reads were removed. Sequencing reads were mapped corresponding to input viruses. These viruses include foot-and-mouth disease virus (FMDV) type O (GenBank accession no. KF112887.1) (A), West Nile virus (WNV) AF404756.1) (B), human rhinovirus 16 (HRV-16) (GenBank accession no. L24917.1) (C), Chikungunya virus (CHIKV) (pJM6-3-CHIKV 181/25-mkate) (D), and Middle East respiratory syndrome coronavirus (MERS) (GenBank accession no. KJ614529.1) (E). Nucleotide coverage depth (NT coverage) is indicated on the *y* axis, and nucleotide (NT) position is indicated on the *x* axis. The genome length for each virus is indicated on the *x* axis, and the percentage of the genome covered greater than 3 nucleotides is indicated. For FMDV, WNV, HRV-16, and CHIKV, data represent material from a single barcode. For MERS, the data shown is a combination of four barcodes generated from the same sample.

We sought to perform the SOP in multiple laboratories and test diverse virus families. Therefore, a standardized version of the SOP was distributed to laboratories that have expertise working with various ssRNA+ virus families (17-19). For proof-of-principle experiments, work was performed with both BSL-2 and BSL-3 ssRNA+ viruses. Representative genomic coverage for FMDV, WNV, HRV-16, CHIKV, and Middle East respiratory syndrome coronavirus (MERS-CoV) obtained from Illumina MiSeq sequencing illustrates that 99.9 to 98% genome coverage was achieved for each virus (Fig. 2A to E). Areas where low coverage was observed include the terminal 5′ and 3′ ends of the genome and large homopolymer regions, such as the poly(C) region present in the 5′ untranslated region (5′-UTR) of FMDV (20). To date, we have sequenced 299 FMDV samples, 49 MERS-CoV samples, 175 WNV samples, 224 HRV (both HRV-14 and -16) samples, and 25 CHIKV samples (representative data from each family are summarized in Table S2 in the supplemental material). An advantage of this system is the ability to create a single library from hundreds of barcoded samples rather than hundreds of libraries, dramatically reducing the time and cost associated with reagents and labor. In cases where the sequences are of sufficient depth, minor variants that differ from the provided reference sequence may be determined using bioinformatic pipelines that integrate CLCbio software (21) and custom software. Representative WNV data (Fig. 2; Table S1) was subjected to minor variant analysis, and we identified 11 base changes from the reference sequence (GenBank accession no. AF404756.1). These changes were a combination of three engineered mutations (positions 8859, 8862, and 8880) (22) and eight additional changes present in the virus stock, serving as a control for our sequencing and analyses.

10.1128/mSystems.00039-15.2 Table S1  Representative output from the SOP data analysis pipeline. Download Table S1, XLSX file, 0.01 MB. Copyright © 2016 Moser et al. 2016 Moser et al. This content is distributed under the terms of the Creative Commons Attribution 4.0 International license.

10.1128/mSystems.00039-15.3 Table S2  Selected sequencing data generated by the SOP for multiple viruses across institutions. All sequencing was performed at JCVI and library construction was prepared at the indicated institutions. Download Table S2, XLSX file, 0.03 MB. Copyright © 2016 Moser et al. 2016 Moser et al. This content is distributed under the terms of the Creative Commons Attribution 4.0 International license.

### The SOP destroys infectious genomic RNA across multiple virus families.

We next sought to evaluate the ability of the SOP to inactivate infectious genomic RNA (gRNA) and infectious virus. High-titer samples of coronaviruses, flaviviruses, alphaviruses, and picornaviruses were processed, and an aliquot of each sample was used to test for infectious virus or infectious viral gRNA (see Materials and Methods and Table 1). Currently, blind passaging of potentially infectious material on permissive cells is a standard assay for infectivity. Negative results from blind passages are often required to remove products out of a BSL-3 facility. This method can detect infectious virus from as little as 1 PFU or from infectious viral gRNA of limited genomes (Table 1). No viral infectivity is present after testing of 309 samples derived from six different ssRNA+ viruses (Table 1). Moreover, no genomic RNA infectivity is present after testing 324 samples. In all cases, positive-control samples confirmed that each cell line clearly detected both viral infectivity and gRNA infectivity. While these results suggest that the SOP completely inactivates infectious material, an additional heat inactivation step was included to further reduce the risk of residual infectious material leaving BSL-3/4 containment. Heat treatment at 72°C for 30 min completely abolishes infectious HRV-16, WNV, and all seven FMDV serotypes (see Table S3 in the supplemental material). Heat inactivation for CHIKV and MERS-CoV was not performed, but our data indicate the ability of the SOP to eliminate infectivity from both enveloped and nonenveloped ssRNA+ viruses. Moreover, high-quality sequence is recovered after heat inactivation of the SISPA products for both WNV and HRV-16 (see Fig. S1 in the supplemental material). It is important to note that for non-SA viruses, the RNA may be removed to the BSL-2 in RNA lysis buffer prior to cDNA generation if approved by the individual’s institutional biosafety committee (IBC).

10.1128/mSystems.00039-15.1 Figure S1  Heating products of the SOP for 72°C for 30 min do not impact sequence quality. Heat inactivation denotes that products of the SOP were treated at 72°C for 30 min prior to sample pooling. No heat inactivation denotes control material from the same sample that was not heated. Insets indicate percent coverage of the genome with heat treatment (red) and without heat treatment (black). Download Figure S1, EPS file, 1 MB. Copyright © 2016 Moser et al. 2016 Moser et al. This content is distributed under the terms of the Creative Commons Attribution 4.0 International license.

10.1128/mSystems.00039-15.4 Table S3  Heat treatment abolishes ssRNA+ viral infectivity. An additional heat inactivation step was included to further reduce the risk of residual infectious material leaving BSL-3/4 containment. Heat treatment at 72°C for 30 min completely abolishes infectious HRV-16, WNV, and all seven FMDV serotypes. Download Table S3, DOCX file, 0.02 MB. Copyright © 2016 Moser et al. 2016 Moser et al. This content is distributed under the terms of the Creative Commons Attribution 4.0 International license.

**TABLE 1:** SISPA products lack both viral and RNA infectivity^a^

Input virus for SISPA^b^	Location^c^	Viral infectivity tests^d^			RNA infectivity tests^e^		
		Cell line	LOD^f^ (PFU) [no. positive/no. tested]	Loss of infectivity (no. positive/no. tested)	Cell line	LOD^f^ (GE) [no. positive/no. tested]	Loss of infectivity (no. positive/no. tested)
HRV-16	JCVI	H1 HeLa	0.01 [0/2]	0/44	H1 HeLa	7.24 × 10^4^ [2/2]	0/44
			0.1 [2/2]				0/13

HRV-14	JCVI	H1 HeLa	NT	0/44	H1 HeLa	NT	0/44

FMDV	USDA	LFBK αvβ6	NT	0/90	LFBK αvβ6	4.23 × 10^5^	0/90
				0/93			0/93
				0/1			
				0/1			

CHIKV	UNC-CH	Vero	0.1 [1/3]	0/25	BHK-21	10^7^ [2/2]	0/25
			1 [3/3]	0/1			0/2

WNV	UW	Vero	0.1 [1/3]	0/3	BHK-21	10^7^ [3/3]	0/3
			1 [3/3]				

MERS-CoV	UMD	Vero	NT	0/3	Vero	NT	0/10

a High-titer samples of representative coronaviruses (MERS-CoV), flaviviruses (WNV), alphaviruses (CHIKV), and picornaviruses (HRV-16, HRV-14, and FMDV) were processed following the SOP, and an aliquot of each sample was used to test for infectious virus or infectious viral genomic RNA (gRNA). Currently, blind passaging of potentially infectious material on permissive cells is the standard for removing products out of a BSL-3 facility. Viral and gRNA infectivity is absent after testing all samples. In all cases, positive-control samples confirmed each cell line clearly detected both viral infectivity and gRNA infectivity.

b The starting material represents viral RNA from at least 1 × 10^5^ PFU. A total of 324 samples were tested for genomic RNA loss of infectivity; 309 samples tested for virus loss of infectivity.

c Abbreviations: JCVI, J. Craig Venter Institute; USDA, U.S. Department of Agriculture; UNC-CH, University of North Carolina at Chapel Hill; UW, University of Wisconsin—Madison; UMD, University of Maryland.

d SISPA products were used to infect the indicated permissive cell line. Three serial passages were performed.

e SISPA products were either electroporated or transfected into the indicated permissive cell line. Three serial passages were performed.

f The limit of detection (LOD) for viral and gRNA infectivity was determined independently from the loss of infectivity testing for each virus. For each loss of infectivity test, a positive control for infectivity (either transfection/electroporation of gRNA or virus infection) was performed in parallel. Abbreviations: NT, not tested; GE, genomic equivalents.

### The SOP completely abolishes gRNA infectivity at multiple steps.

**FIG 3:**
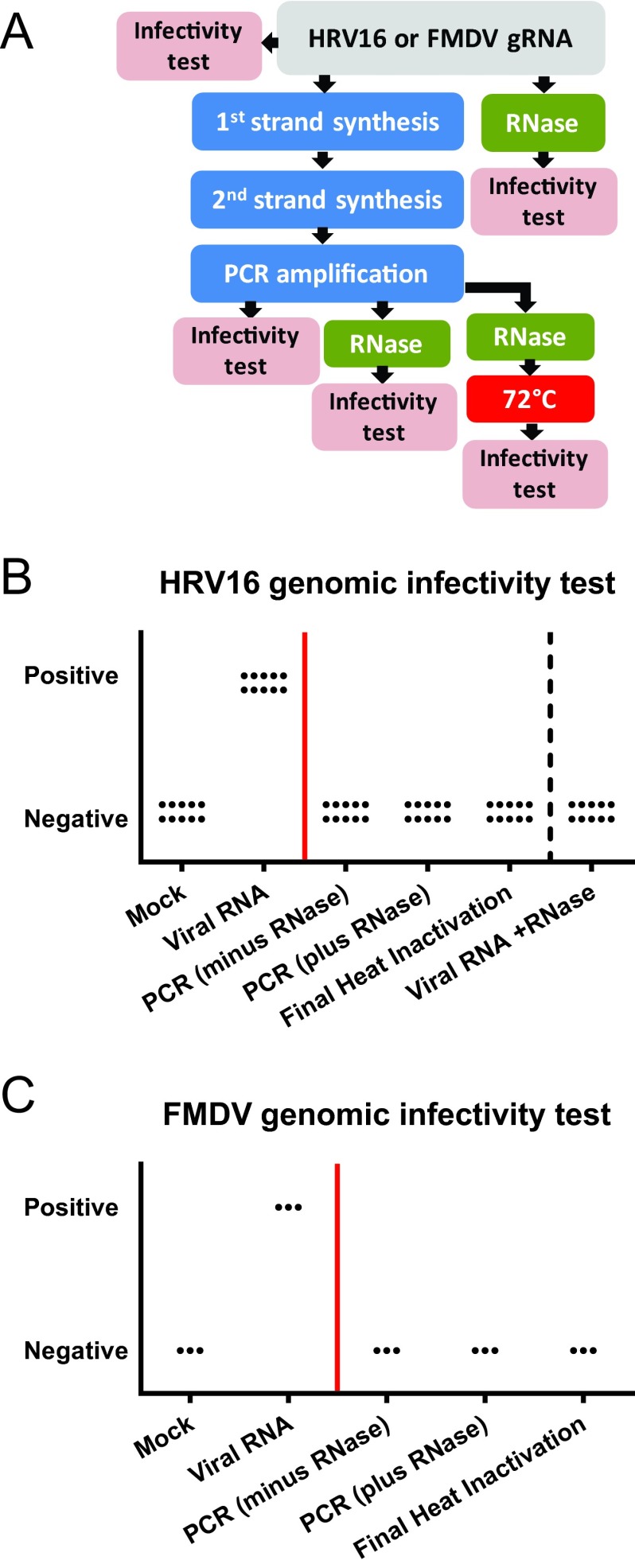
Performing the SOP with both HRV-16 and FMDV to identify where loss of genomic RNA infectivity occurs. (A) Flow chart depicting a test for HRV-16 or FMDV loss of RNA infectivity. Briefly, 60 tubes of HRV-16 gRNA were subject to six different conditions in replicates of 10. The SOP was performed, and a subset was purified at each step and tested for the presence of infectious RNA over three blind passages on H1 HeLa cells. For FMDV, viral RNA, intermediates, or the final SOP products were electroporated into LFBK αvβ6 cells. (B) Results of infectivity testing with HRV-16. Each symbol represents the value for an individual sample. Samples to the right of the red line highlight steps where all samples tested had no detectable infectious HRV-16 genomic RNA. The “Viral RNA +RNase” group demonstrates the RNase treatment is sufficient to inactivate all infectious gRNA. (C) Results of infectivity testing with FMDV as outlined in panel A. Each symbol represents the value for an individual sample. Samples to the right of the red line highlight steps where all samples tested had no detectable infectious FMDV genomic RNA.

Our initial results demonstrate that the SOP removes all gRNA infectivity from starting material derived from high-titer ssRNA+ viral stocks. We next initiated experiments to define a specific step in the SOP where gRNA infectivity is abolished, working with the small RNA virus HRV-16, which is highly infectious when introduced into permissive cells (23). We first determined that the limit of detectable RNA infectivity for HRV-16 occurs in the range between 0.1 and 0.01 PFU on H1 HeLa cells (Table 1). Next, gRNA from HRV-16 (1 × 10^6^ PFU equivalents per sample) were subjected to either the entire SOP or stopped at intermediate steps (diagrammed in Fig. 3A). Briefly, 10 replicates for select steps in the SOP were transfected into H1 HeLa cells. First, purified HRV-16 RNA from 10^6^ PFU was transfected into H1 HeLa cells prior to initiating the SOP (positive control for transfection). Second, the SOP was stopped after PCR amplification, and the PCR products were purified. Third, the SOP was used to generate PCR products, which were treated with 2 µl of an RNase cocktail and purified. Fourth, the final products from the SOP were heat inactivated (which encompasses the entire SOP). As a control for the efficacy of the RNase cocktail, RNA from 10^6^ PFU of HRV-16 was treated with the amount of RNase from the SOP. Material from each group was then transfected into H1 HeLa cells and subjected to three blind passages to demonstrate that infectivity was destroyed.

Results clearly indicate that all infectious gRNA is removed at the step of PCR (Fig. 3B), likely resulting from the temperature cycling conditions. However, we cannot rule out the contribution of RNase H in the cDNA synthesis step to the degradation of infectious gRNA (described in the supplemental SOP [Text S1 in the supplemental material]). Moreover, the subsequent RNase step and final heat treatment for 30 min at 72°C serve as additional safety checkpoints. Our data suggest that both the PCR step and the downstream RNase cocktail inactivate gRNA from at least 1 × 10^6^ PFU equivalents for HRV-16, proving that the ability of the SOP to inactivate gRNA is extremely robust. To build on the data obtained with HRV-16, a similar approach was followed with FMDV (Fig. 3). Corroborating the HRV-16 results, FMDV gRNA infectivity is abolished post-PCR in the absence of the RNase cocktail (Fig. 3C). These two experiments highlight that the SOP efficiently inactivates all infectious gRNA at an early step in the SOP and that subsequent steps serve as additional fail-safes for inactivation. This is true for both FMDV and HRV-16, and supports data in Table 1 which clearly demonstrate that the SOP abolishes gRNA infectivity across diverse virus families.

### Limiting dilution experiments with the SOP identify comparable sensitivities between the Illumina MiSeq and HiSeq platforms.

**FIG 4:**
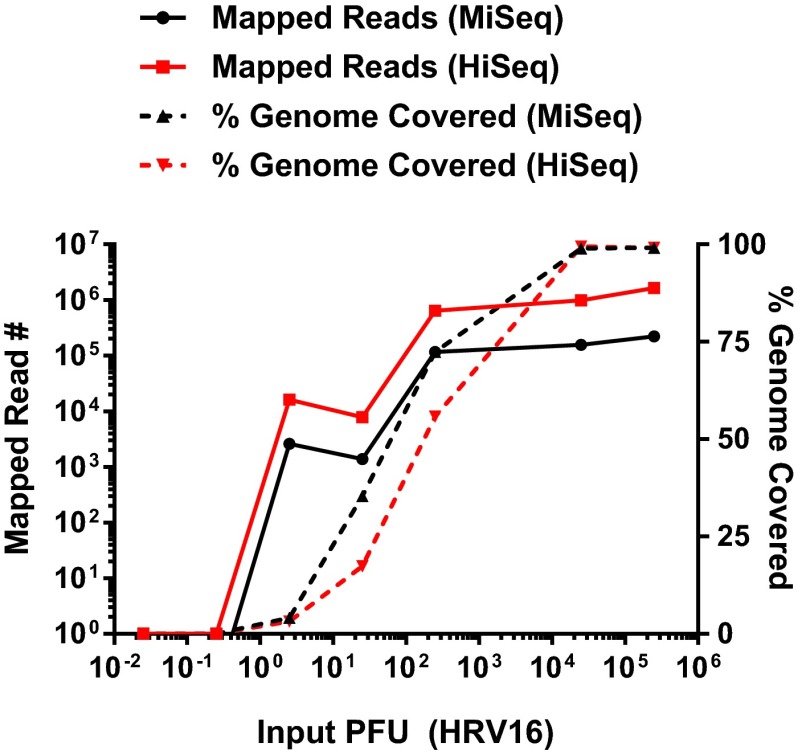
Defining the sensitivity of the SOP on Illumina MiSeq and HiSeq platforms. RNA from serial 10-fold dilutions of an HRV-16 virion stock was treated according to the SOP. Samples were pooled and sequenced on an Illumina MiSeq or HiSeq platform. The left *y* axis denotes the number of reads mapped to the HRV-16 reference genome, the right *y* axis denotes the percentage of the reference genome covered, and the *x* axis denotes the input PFU for each reaction. The solid black line demonstrates that sequencing reads were detected between 1 and 10 PFU on the MiSeq platform. A similar sensitivity is obtained on the HiSeq platform, as denoted by the solid red line. The corresponding percentage of the HRV-16 genomic coverage from each platform is denoted by a dashed black line (MiSeq) and a dashed red line (HiSeq). The slight enhancement of genomic coverage on the MiSeq platform, despite the fewer number of sequence reads, results from the longer read length on the MiSeq platform (300 nucleotides [nt]) over the HiSeq platform (100 nt), as sequencing capacity is in excess at all dilutions.

To determine the sensitivity of the SOP across multiple sequencing platforms, we sequenced identical libraries generated from the SOP with both MiSeq and HiSeq. Representative data from a serial dilution experiment shows that the limit of detection (LOD) for HRV-16-specific sequencing reads was approximately 2.5 PFU (Fig. 4). The HiSeq platform produces an average of 5- to 7-fold-more sequencing reads for each sample. However, these additional reads do not improve the overall genome coverage nor the limit of detection (Fig. 4). Therefore, we conclude that the MiSeq platform is able to sequence all viral reads present in each library and that the HiSeq platform simply derives more sequences from the same starting material.

### Evaluating the sensitivity of the SOP from purified virions, purified viral genomic RNA, and mixed samples.

**FIG 5:**
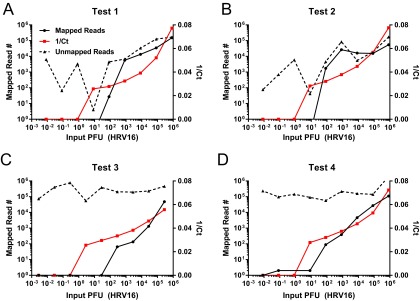
NGS on SOP-generated HRV-16-specific sequence from pure and mixed samples is slightly less sensitive than quantitative real-time RT-PCR (qrRT-PCR). Four independent tests were conducted to determine the sensitivity of the SOP. Test 1 detects HRV-16 sequence from dilutions of purified virus. Test 2 detects HRV-16 sequence from dilutions of genomic RNA. Test 3 detects HRV-16 sequence from dilutions of virus spiked into H1 HeLa cells. Test 4 detects HRV-16 sequence from genomic RNA dilutions spiked into total HeLa cell RNA. A ribosomal removal step was performed for tests 3 and 4 prior to the initiation of the SOP. For each sample, a fraction of the RNA used to initiate the SOP was subjected to qrRT-PCR analysis. (A to D) HRV-16-specific reads obtained by MiSeq (black solid lines) are plotted on the left *y* axis and the cycle threshold (Ct) values are plotted on the right *y* axis (red lines). Sequencing reads not mapping to the HRV-16 reference are also indicated (black dashed lines). Corresponding HRV-16 input PFU values are plotted on the *x* axis. (A) The limit of detection (LOD) for test 1 in this experiment is between 10^1^ and 10^2^ input PFU. The corresponding LOD by qrRT-PCR is approximately 10-fold greater (10^0^ to 10^1^ input PFU). (B) The LOD for test 2 in this experiment is between 10^1^ and 10^2^ input PFU. The corresponding LOD by qrRT-PCR is approximately 10-fold greater (10^0^ to 10^1^ input PFU). (C) The LOD for test 3 in this experiment is between 10^2^ and 10^3^ input PFU. The corresponding LOD by qrRT-PCR is approximately 100-fold greater (10^0^ to 10^1^ input PFU). (D) The LOD for test 4 in this experiment is between 10^1^ and 10^2^ input PFU; however, single reads are detected down to an input of 10^−1^. The corresponding LOD by qrRT-PCR is approximately 10-fold greater (10^0^ to 10^1^ input PFU) when individual HRV-16 reads are not considered and approximately 10-fold less sensitive when individual HRV-16 reads are considered.

We next sought to further define the SOP limits of detection on a MiSeq platform and directly compare this sensitivity to sequence-specific quantitative real-time reverse transcriptase PCR (qrRT-PCR). Separate experiments with dilutions of HRV-16 virions and HRV-16 gRNA in both pure (cell-free supernatant containing virions) and mixed (cellular RNA spiked with HRV-16) samples were performed. For test 1 (Fig. 5A), a virion dilution series precedes RNA extraction, followed by the SOP. For test 2 (Fig. 5B), virion RNA was extracted from a high-titer stock, followed by serial dilution, and aliquots from each dilution were subjected to the SOP. For test 3 (Fig. 5C), virion dilutions described in test 1 were mixed with HeLa cells. Total RNA was extracted from each tube, and rRNA was removed before proceeding to the SOP. For test 4 (Fig. 5D), aliquots from each dilution of viral RNA described in test 2 were added to 1 µg of total HeLa cell RNA. rRNA was removed, and the material was subjected to the SOP.

Representative MiSeq data (black line) from each test is displayed in Fig. 5A to D as the number of HRV-16-specific sequencing reads per sample. Prior to initiating the SOP, an aliquot of diluted RNA from each sample was analyzed by qrRT-PCR to determine the input viral RNA copy number (red line). This input value provides a comparison for the sequence read output. The approximate qrRT-PCR and MiSeq LODs for this experiment are as follows: qrRT-PCR LOD of 8.33 PFU and MiSeq LOD of 83.3 PFU in test 1, qrRT-PCR LOD of 8.33 PFU and MiSeq LOD of 83.3 PFU in test 2, qrRT-PCR LOD of 2.78 PFU and MiSeq LOD of 162 PFU in test 3, and qrRT-PCR LOD of 8.33 PFU and MiSeq LOD of 27 PFU in test 4. As expected, qrRT-PCR is more sensitive than MiSeq sequencing is. However, the SOP has the advantage over qrRT-PCR of requiring no *a priori* knowledge of the sequence of interest and has the benefit of providing sequence information from both known and unknown samples.

### Evaluating the sensitivity of the SOP in WNV-infected cells and tissues.

**FIG 6:**
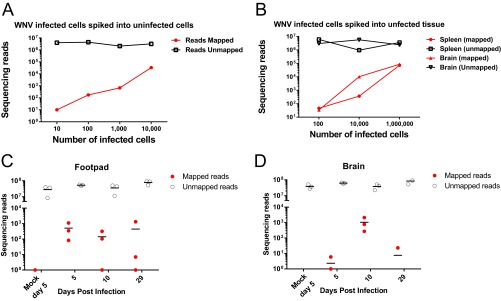
The SOP can detect WNV infection *in vitro* and *in vivo*. (A and B) Infected WNV cells were spiked into uninfected cells (A) or uninfected tissues (B), libraries were prepared on RNA according to the SOP, and the libraries were examined by Illumina MiSeq. (C and D) Footpad (C) and brain tissue (D) from WNV-infected mice were analyzed at 5, 10, and 29 days postinfection for WNV-specific sequence reads by Illumina HiSeq. (A) Data representing the ability of the SOP to identify WNV-specific reads from limiting dilutions of WNV-infected Vero cells spiked into uninfected 293T cells. Mapped and unmapped reads from each sample are displayed. (B) The SOP identifies WNV-specific reads from limiting dilutions of WNV-infected Vero cells spiked into uninfected mouse tissues (spleen and brain). Mapped and unmapped reads from each sample are shown. (C) WNV was detected in the footpad RNA of mice prepared according to the SOP at the indicated times postinfection. (D) WNV-specific reads can be detected from brain tissue RNA of mice at the indicated times postinfection. For panels C and D, three mice per group were analyzed, and WNV-mapped and unmapped reads are shown.

We further evaluated the ability of the SOP to detect WNV in the context of cells or whole tissue. Limiting dilutions of WNV-infected cells were spiked into uninfected cells (Fig. 6A) or uninfected tissues (Fig. 6B). Our method detects WNV-specific reads from as few as 10 infected cells spiked into uninfected cells (Fig. 6A) and as few as 100 infected cells spiked into uninfected tissues (Fig. 6B). Another experiment was designed to determine the ability of the SOP to detect WNV-specific sequencing reads in both acutely and persistently infected mouse tissues. For this study, the SOP was performed to generate libraries from the footpad (site of inoculation) and the brain (target organ of WNV) at days 5, 10, and 29; both tissues support viral production with infectious virus present for at least 1 month after inoculation (18, 24). Samples were pooled and sequenced on eight HiSeq lanes to ensure sufficient sequencing depth for a proper analysis, yielding between 20 and 90 million reads per sample. Viral loads peak in the footpad at day 5 and diminish by day 29 (Fig. 6C), while peak brain titers occur at day 10 postinoculation (Fig. 6D). Furthermore, virus-specific reads were detected in the brain of one animal at 29 days postinfection, which underscores the power of the SOP to detect low levels of virus in a natural infection.

### The SOP can accurately identify “unknown” samples.

**FIG 7:**
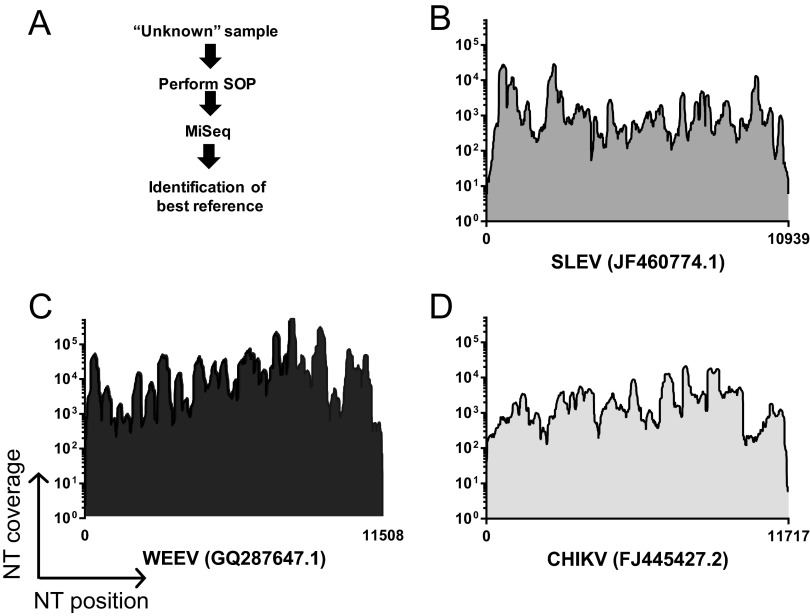
The ability of the SOP to sequence and identify unknown samples. (A) High-titer viral stocks were subjected to the SOP, anonymized, and shipped to JCVI for sequencing and data analysis. Samples were pooled and sequenced by Illumina MiSeq. Data from each corresponding sample were put into *de novo* assembly, and large contigs (>500 bp) were used to identify the best full-length viral genome references by nucleotide BLAST search against the NT database. Raw data were then mapped onto the best available reference genome. (B) Mapping coverage of an unknown sample against the selected genome for St. Louis encephalitis virus (SLEV). (C) Mapping coverage of an unknown sample against the selected genome for Western equine encephalitis virus (WEEV). (D) Mapping coverage of an unknown against the selected genome for Chikungunya virus (CHIKV). In panels B to D, nucleotide coverage depth is indicated on the *y* axis, and genomic position, with the length of each genome indicated as well as the best available reference genome, is indicated on the *x* axis.

To test the ability of the SOP to recover viral sequence from an unknown sample source, three viral stocks were subjected to the SOP, anonymized, and shipped to the J. Craig Venter Institute (JCVI) for sequencing and data analysis in a blind manner (Fig. 7A). Raw data underwent *de novo* assembly, and large contigs (>500 bp) were used to identify the best full-length viral genome references by a nucleotide BLAST search. Sequencing reads were then mapped onto the selected reference genome, and mapping coverage was determined (Fig. 7). For St. Louis encephalitis virus (SLEV), Western equine encephalomyelitis virus (WEEV), and Chikungunya virus (CHIKV), we obtained more than 98% genome coverage, clearly demonstrating the ability of the SOP to rapidly identify ssRNA+ viruses from samples without prior information. In addition, we successfully identified an “unknown” sample, which was a mix of four different viral RNAs (WNV, SLEV, WEEV, and CHIKV) (data not shown).

## DISCUSSION

The core objective of a biosafety program is the containment of potentially harmful biological and infectious agents. Standardization of lab practices at all BSLs are defined (7), but the transfer of potentially infectious material out of high-containment facilities is very challenging and is sometimes the source of containment breaches. Likewise, during outbreak situations, the need to handle samples containing potentially infectious viral genomes (or genetic material falling under SA regulations) under high containment may significantly hinder public health responses. In this report, we demonstrate the development of a robust SOP for generating high-quality cDNAs from ssRNA+ viruses, which can be used in highly parallel processes to quickly and safely remove samples from BSL-3/4 containment for subsequent genomic sequencing or other similar procedures in BSL-2 laboratories. Protocols to generate NGS sequencing libraries for ssRNA+ viruses do exist. For example, a recent report (20) describes a method for FMDV whole-genome sequencing, but it was not proven to destroy infectious material. In addition to complete genome coverage, our data confirm that full-length RNAs, cDNAs, or dsDNAs from these processes cannot be used to recover/rescue infectious viruses. We demonstrate that this SOP completely inactivates viruses from diverse virus families classified as SAs or considered high-consequence pathogens. The SOP removes infectious genomic RNAs and simultaneously amplifies barcoded cDNAs that cannot be used to rescue/recover viruses (Table 1 and Fig. 3). The SOP also generates high-quality cDNA libraries, and the resulting NGS data covers almost entire ssRNA+ viral genomes from samples tested (Fig. 2 and 4). The SISPA method is unable to fully define viral genomic termini, since the method utilizes random hexamer priming. Therefore, additional protocols, such as rapid amplification of cDNA ends (RACE) are required for this sequence information. The procedure is sequence independent and can be utilized on both known and unknown viruses (new/emerging), regardless of prior genome information. Sequence independence also enables simultaneous identification of multiple pathogens in a sample (e.g., coinfections) or even modified, manufactured pathogens. The described strategy is efficient, scalable, and inexpensive and requires only reagents and equipment that are commonly used in BSL-3/4 laboratories, whereas NGS sequencing equipment is expensive and difficult to maintain, especially within high-containment laboratories.

This SOP builds on the established SISPA method (13, 14) to create small overlapping fragments that are barcoded (i.e., uniquely tagged) by the addition of nucleotides to the termini. Our barcoding system has many advantages. (i) The barcoded DNAs cannot be used to recover/rescue viruses, so they can be safely transferred to BSL-2 sequencing centers onsite or shipped to large-scale sequencing centers. (ii) Our system allows for the PCR amplification of the genomic material (thus, limited amounts of sample or low copy number can yield whole genomes). (iii) It can be scaled up for high-throughput sequencing of hundreds of samples simultaneously, which can dramatically reduce the cost and effort required for NGS library construction.

Collaboratively, the SOP has been performed more than 1,000 times in multiple laboratories to demonstrate that full-length genomic sequences can be obtained, and it can be used to detect very small amounts of viral nucleic acid approaching the sensitivity of qrRT-PCR. The strategy can be applied to diverse starting material such as cell culture supernatants, infected-cell monolayers, and animal tissues. While much of the representative data shown was derived from Illumina MiSeq, the SOP can be applied to multiple platforms, including Illumina HiSeq and Ion Torrent Personal Genome Machine (PGM). The PGM provides a rapid turnaround time, which allows for viral sequence data to be obtained in hours compared to longer times required for Illumina platforms. An interesting result from this study was the observation that both the Illumina MiSeq and HiSeq platforms have similar limits of detection on identical NGS libraries (Fig. 4). This is likely because the MiSeq platform sequences most virus-specific molecules in the library, and the HiSeq platform only adds more reads from the same starting material. It is important to note that the HiSeq platform is still relevant for host gene expression analysis, since additional sequencing depth is critical to provide adequate coverage across all mammalian mRNAs. We are also able to directly compare the sensitivity of the SOP to sequence-specific quantitative qrRT-PCR methods (Fig. 5). While qrRT-PCR is more sensitive, it requires prior knowledge of the sequence of interest, while SISPA does not. Further, we can detect HRV-16-specific sequence from both limiting amounts of HRV-16 virion RNA and from samples where the majority of the sequence data are host derived. We demonstrate the ability to detect low levels of WNV-specific sequence from persistently infected mouse tissues as late as 29 days postinfection (Fig. 6). Future studies will optimize the SOP to improve assay sensitivity to further reduce sequencing cost in samples with limited starting material. However, these data illustrate the value of this protocol for virus discovery and/or forensic analyses.

Extensive effort was directed toward proving the SOP abolishes viral and genomic infectivity across virus families. Our experimental design is sufficient to state with high confidence that the SOP abolishes infectivity because of the following. (i) The input infectious dose for each sample is high. (ii) The limit of detection for each infectivity test is low. (iii) The sample size is sufficiently high. It is important to note that to assign a confidence interval for this assay, there must be some failures or variability in the final outcome (e.g., cultures positive for infectivity during loss of infectivity testing). Our processing of all samples since the initiation of the experiment has a success rate of 100% (Table 1). The ability to demonstrate that all RNA infectivity is lost after the PCR step for both HRV-16 and FMDV (Fig. 3) strongly suggests that a final RNase step serves as an additional safeguard to remove infectious RNA. Finally, the heat step of 72°C for 30 min ensures no residual infectivity due to lab contamination is present prior to transfer from BSL-3 to BSL-2 (see Table S3 and Fig. S1 in the supplemental material). However, this step is considered fail-safe, as we demonstrate that infectivity is abolished after the PCR step (Fig. 3).

Applying NGS to sequencing RNA viruses represents an unparalleled capacity to generate large amounts of sequence data, which can be used to identify consensus sequences as well as minor sequence variants, or quasispecies, present in a viral population (25). Limited FMDV population diversity studies explore both intrasample variation during serial passage and the presence of FMDV quasispecies within an infected animal (26, 27); therefore, the described SOP is especially promising for FMDV genomic analyses. To overcome the cost and time limitation of using conventional NGS protocols for FMDV studies in large data sets, a previous study combined NGS and Sanger data with sequences available in public databases to study microevolutionary processes of FMDV populations at multiple scales (28). A protocol to generate consensus level genome sequences for FMDV and a few other positive-sense polyadenylated RNA viruses has been reported (20), but it was exclusively tested on an Illumina MiSeq platform. In contrast, our SOP provides extra versatility and cost efficiency, as it has been adapted to multiple sequencing platforms, and it allows analysis of up to 288 barcoded samples in a single run. Therefore, the scalability and cost-efficiency of the SOP described here make it a promising approach to examine quasispecies from large data sets, and it can provide sufficient sequencing depth to examine minor variants from high-titer stocks (see Table S1 in the supplemental material). Consistent with previous observations (20), we also found lowest coverage in highly structured or repetitive regions of the 5′-UTR. Further optimization of the SOP is required to investigate low-level viral populations or structurally difficult genomic areas from all starting material (25).

Rapidly evolving NGS technologies have improved our ability to discover novel viral sequences across diverse sample types (29, 30). Transcriptomics, analyzed historically through microarray and more recently by NGS, represent another approach to identify both known and novel viral sequences in a given sample (reviewed in reference 31). Alternative virus discovery approaches have utilized degenerate primers for specific virus families, virus-specific probes (32), and random primers for signal amplification and subsequent NGS library construction (13, 14). Our SISPA approach couples a barcode sequence and random hexamer in a single oligonucleotide and requires no prior knowledge of the sample composition (Fig. 7). Moreover, HRV-16 data demonstrate the ability to obtain HRV-16-specific reads from gRNA corresponding to fewer than 10 PFU (Fig. 4). Importantly, the final products of the SOP are small dsDNA fragments flanked by sequence barcodes and therefore can be safely and stably shipped to a sequencing facility in the absence of a cold chain.

In summary, this universal SOP for cDNA production that reproducibly inactivates infectivity of BSL-3/4 viruses will speed the transfer of nucleic acids from high-containment laboratories to BSL-2 laboratories where NGS can be rapidly performed. This SOP is suitable for any pathogen, because it is sequence independent and can be utilized on both known and unknown agents and will simultaneously identify multiple agents. Finally, our collection of data suggests that our procedure can be implemented and easily validated by institutional biosafety committees across research laboratories.

## MATERIALS AND METHODS

### Viral sequencing.

Prior to the generation of sequence data, an SOP was established for work at each institution. These institutions include the J. Craig Venter Institute (JCVI), University of Wisconsin—Madison, Plum Island Animal Disease Center (PIADC), University of Maryland School of Medicine, and University of North Carolina—Chapel Hill. A detailed, standardized protocol describing all procedures and reagents is supplied in the supplemental material. Briefly, total RNA was extracted from viral stocks, virus-infected cells, or virus-infected tissues. Random hexamer oligonucleotides coupled with unique barcodes (denoted BC-N6) were used for first-strand cDNA (Life Technologies) and second-strand DNA (New England Biolabs) synthesis. The first-strand cDNA synthesis step also includes treatment with RNase H (catalog no. M0297S; New England Biolabs) (see Text S1 in the supplemental material). A second primer, specific for each barcode but lacking a random hexamer, was then used to generate PCR fragments ranging in size from approximately 300 to 1,000 bp (Life Technologies). An example of a BC-N6 primer sequence is 5′-TAGTACACTCTAGAGCACTANNNNNN-3′, and the corresponding PCR primer sequence is 5′-TAGTACACTCTAGAGCACTA-3′. In order to overcome the problems associated with low diversity in the first cycles of Illumina reads (33), we modified the SISPA protocol by adding either 3 N’s or 4 N’s to the 5′ ends of the SISPA PCR primers for each barcode, and using an equimolar combination of these modified primers during the PCR amplification step of the protocol. A list of BC-N6 and PCR primer sequences has been described previously (15). All SOP products were treated with an RNase cocktail (Life Technologies) and purified (Qiagen). A subset of samples were subjected to heat inactivation at 72°C for 30 min. All BSL-3 samples that required the loss of virus and RNA infectivity were subjected to testing consistent with the specific institutional biosafety standards. Final products of the SOP were shipped to JCVI, pooled, and sequenced on the Illumina MiSeq (two 300-bp paired end [PE] sequencing reads), Illumina HiSeq (two 100-bp PE), Illumina NextSeq (two 150-bp PE), or Ion Torrent (318 Chip 400-bp kit) platforms.

### Postsequencing genomic analyses.

A combination of JCVI-developed and CLCbio-developed command line tools were used to process read data from all NGS runs. Duplicate reads were removed, reads were demultiplexed based on each sample’s specific barcode sequence, and reads were both sequence trimmed to remove the barcode and random hexamer and quality trimmed to remove low-quality bases. SISPA barcodes are designed to have a minimum edit distance of 5 between any two barcodes, such that during demultiplexing, two errors may be allowed when searching for barcode sequences at the termini of reads. For barcode demultiplexing, we used bespoke software available from http://sourceforge.net/projects/deconvolver/ to identify SISPA barcode sequences, bin and trim the barcode and random hexamer, but other software packages may be used as well. For samples with a known input sequence, reads were mapped to a prespecified reference sequence. For samples with an unknown input sequence, reads were *de novo* assembled, and the resulting contigs longer than 500 nucleotides (nt) were used to search for the most appropriate reference sequences available in GenBank, using NCBI’s BLASTN against the NT database, with a filter for viruses (taxonomy identifier [ID] [taxid]10239). Once the closest references were chosen, reads were mapped to the selected references. For all analyses, the number of reads that mapped to the references, the percent genome coverage from these mappings, and the number of unmapped reads were determined. When sufficient sequence depth was available, single nucleotide polymorphisms (SNPs) were determined using JCVI custom software. This pipeline applies statistical tests to minimize false-positive SNP calls that could be caused by the types of sequence-specific errors (SSE) that may occur in Illumina reads previously identified and described (21). Once a minimum minor allele frequency threshold and significance level are established by the user, the number of minor allele observations and major allele observations in each direction and the minimum minor allele frequency threshold are used to calculate a *P* value based on the binomial distribution cumulative probability, and if the *P* values calculated in each of the two sequencing directions are both less than the Bonferroni-corrected significance level, then the SNP call is accepted. For our analyses, we used a significance level of 0.05 (Bonferroni corrected for tests in each direction to 0.025), and a minimum minor allele frequency threshold of 3%.

### FMDV methods. (i) FMDV stocks and viral infectivity studies.

For FMDV, transcribed RNA from FMDV A24 Cruzeiro infectious clone (34) and LFBK αvβ6 cells were used to assess the presence or absence of viral infectivity (8). LFBK αvβ6 cells were used to assess the presence or absence of viral infectivity as previously described (8). Cells were propagated using Dulbecco’s modified Eagle medium (DMEM) supplemented with 10% fetal bovine serum (FBS) and antibiotics and incubated at 37°C in 5% CO_2_ (35). Briefly, T25 flasks seeded with LFBK αvβ6 cells for 48 h were rinsed and inoculated with SISPA products in 2 ml of serum-free medium. After 1 h of adsorption, 3 ml of medium with 1% serum were added to the flasks, and the flasks were incubated for 72 h at 37°C on a rocking platform. Samples in which no infectivity was observed were amplified through three blind passages.

### (ii) FMDV RNA infectivity studies.

BHK-21 cells (baby hamster kidney cell strain 21, clone 13; ATCC CCL10) were used for electroporations. Cells were maintained in minimal essential medium (MEM) containing 10% tryptose phosphate broth, 10% calf serum, and 1% antibiotics and nonessential amino acids (Gibco-BRL/Invitrogen). SISPA products or FMDV RNA were electroporated as previously described (36). The electroporation products were subsequently passaged on LFBK αvβ6 cells as described above to assess viral infectivity for a total of three blind passages. To determine the limit of recovery for our system, we electroporated serial dilutions of quantified recombinant wild-type A24 RNA (37). The electroporation products were passaged a minimum of three times on LFBK αvβ6 cells, with infectivity visually checked daily.

### (iii) RNA isol ation, cDNA synthesis, and FMDV detection by qPCR.

Total RNA was isolated from supernatants of cell lysates previously electroporated with viral RNA, cDNA, or SISPA using either the Qiagen RNeasy kit (Valencia, CA) or the MagMax-96 viral RNA isolation kit (Ambion) on a KingFisher magnetic particle processor (Thermo Scientific) for high-throughput analysis. RNA was treated with RNase-free DNase I (Sigma-Aldrich). Superscript III reverse transcriptase (RT) (Life Technologies) was used for cDNA synthesis following the manufacturer’s instructions. cDNA was diluted 1:100 and run in duplicate following as described previously for FMDV (38) using Path-ID quantitative PCR (qPCR) reagents (Applied Biosystems) in a 7500 thermocycler (Applied Biosystems). Samples were considered positive when threshold cycle(*C_T_*) values were <40.

### (iv) FMDV thermal stability study.

A virus stock of FMDV A24 was prepared at 2 × 10^5^ PFU/ml. Quadruplicate tubes were heated at 72°C for 5 min, 72°C for 30 min, and 72°C for 60 min or 4°C for 60 min (control). Samples were then stored at −70°C for further titration by plaque assay (39).

### WNV methods. (i) WNV stocks and RNA isolation.

Two stocks of WNV were used in the studies. One stock (WNV 3356K) was a biological isolate. Another stock (FL-WNV) was derived from an infectious cDNA clone of WNV 3356K, as previously described (22). FL-WNV contains three silent mutations engineered into the infectious clone and, thus, can be differentiated from WNV 3356K (22). Titers of virus stocks were determined on African green monkey kidney cells (Vero; ATCC CCL-81).

RNA was isolated from samples with the Qiagen RNeasy kit according to the manufacturer’s instructions. The initial sample volume was 100 µl, and isolated RNA was eluted in 30 µl RNase-free water. All animal studies that resulted in tissue sample harvest were approved by IACUC at the University of Wisconsin—Madison. RNA was isolated from tissues using a combination of Trizol reagent (Life Technologies) and the Qiagen RNeasy kit. Tissue sections (3 mm by 3 mm by 3 mm) from the brains, spleens, and livers of mice were placed in 0.5 ml Trizol with a 0.18-inch diameter steel ball and homogenized with a Qiagen mixer mill for 4 min at 24 cycles/s. The process was repeated as needed until complete homogenization was observed. The samples were incubated for 10 min at room temperature (rt) prior to the addition of 100 µl of chloroform. Samples were mixed, incubated for 2 min at rt, and centrifuged for 12,000 × *g* for 15 min at 4°C. The aqueous fraction was transferred to a fresh tube, mixed with 300 µl of cold 70% ethanol, and added to an RNeasy column, and RNA was extracted following the manufacturer’s instructions. rRNA was depleted from 2 µg of total RNA using the RiboZero Magnetic kit (Epicentre, Madison, WI) according to the manufacturer’s instructions.

### (ii) WNV viral infectivity studies.

RNA was isolated as described above, and SISPA libraries were generated according to the universal standard operating procedure. One to three SISPA libraries were pooled, and 15 µl of the pool was added to 1 ml virus diluent (MEM plus 1% FBS). A negative-control sample (diluent alone) and a positive-control sample containing 100 PFU were prepared in parallel. The 1-ml samples were inoculated into a T25 flask of Vero cells (18) and incubated at 37°C and 5% CO_2_ for 1 h, rocking every 15 min. Five milliliters of complete medium (MEM plus 10% FBS) was added to each flask, and the cells were incubated at 37°C and 5% CO_2_ for 4 days. Monolayers were monitored daily for cytopathic effect (CPE). If CPE was apparent, aliquots were collected and frozen at −80°C, and the sample was no longer passaged. If no CPE was present 4 days postinoculation (dpi), 1 ml of supernatant from each flask was inoculated onto fresh Vero cells, initiating the infection process again for a total of three passages. To determine the limit of detection for infectious virus, 10-fold dilutions of WNV (10^2^ to 10^−3^ PFU) were inoculated onto Vero cells and subjected to four rounds of blind passaging as described above. The experiment was run in triplicate. CPE was evident in all cells infected with at least 1 PFU by the second passage. Mild CPE developed in one flask infected with 0.1 PFU at the fourth passage. No CPE was observed in cells infected with less than 0.1 PFU.

### (iii) WNV RNA infectivity studies.

The presence of infectious WNV RNA in SISPA libraries was assayed by electroporation of BHK-21 cells followed by serial passage on Vero cells. BHK-21 cells were trypsinized, washed, and resuspended in PBS at a concentration of 1.25 × 10^7^ cells/ml in preparation for electroporation. Cells (0.8 ml) were mixed with 15 µl of water, 15 µl of SISPA pool, or 10 µl of water plus 5 µl of WNV RNA (isolated from virus stock as described above) in a 0.4-mm-gap electroporation cuvette. The cell mixture was pulsed in an electroporator (BioRad, Richmond, CA) three times at 850 V for 0.4 s with 3-s rests. The cells were incubated for 10 min at rt before they were transferred to a flask containing 15 ml warmed complete medium. The cells were grown at 37°C and 5% CO_2_ and were observed daily for CPE. After 4 days, 1 ml of supernatant from each flask was inoculated onto Vero cells, and the samples were serially passaged as described above.

To determine the limit of rescue for infectious RNA in our system, BHK-21 cells were mixed with 10-fold dilutions of WNV RNA (10^5^ to 10^8^ genomic equivalents [GE]) of WNV and electroporated in triplicate. GE were calculated from isolated viral RNA using real-time RT-PCR (40). Cell supernatants were inoculated onto Vero cells and subjected to four blind passages. CPE rapidly developed in cells that were transfected with 10^7^ or 10^8^ GE, while no CPE developed in cells transfected with less than 10^7^ GE. SISPA libraries were considered free from infectious virus or RNA if no CPE was observed by the end of the third passage on Vero cells. As a secondary test for infectious material, 100 µl of supernatant from the final passage of each sample was inoculated onto Vero cells in duplicate. The infection was allowed to continue for 2 days, at which time monolayers were fixed and examined for virus antigen by immunofluorescence. Monolayers were stained with mouse hyperimmune ascitic fluid (CDC; 1:100) followed by fluorescein isothiocyanate (FITC)-conjugated goat anti-mouse (KPL; 1:100) and visualized with a Nikon Eclipse TS100 fluorescence microscope. Viral antigen was observed in all wells inoculated with positive-control WNV samples but was absent in all wells inoculated with negative-control or SISPA library supernatant.

### HRV-16 and HRV-14 methods. (i) HRV viral stocks and quantitative PCR.

The pWR3.26 molecular clone was obtained from the ATCC to rescue HRV-14 viral stocks, while the pR16.11 plasmid was obtained from the ATCC to generate HRV-16 viral stocks (GenBank accession no. L24917). Supernatants containing infectious virus for both HRV-16 and HRV-14 were isolated and aliquoted. The GenBank reference sequence for HRV-16 was used to design a quantitative reverse transcriptase PCR (qRT-PCR) TaqMan primer/probe set: TaqMan forward primer, 5′-GGTTAAATGGATGTTAAGAATTATATCAGCTATGGTTATA-3′; TaqMan reverse primer, 5′-CATCCAATCAGTGTTAAAGTGGCAAT-3′; TaqMan probe, 6-carboxyfluorescein (FAM)-CAGATCCGCAAACAAT. To isolate RNA, supernatant after each passage was frozen at −80°C and subsequently subjected to RNA extraction using a Qiagen RNeasy kit. RNA was treated with RNase-free DNase I. Reverse transcription was performed with Superscript III reverse transcriptase (Invitrogen). As a negative control to monitor contamination, water was used in place of Superscript III reverse transcriptase, and in each case, reverse transcription was performed with oligo(dT) priming. cDNA was diluted to 1:100 and run in duplicate using the TaqMan custom HRV-16 custom primers and probe on the Applied Biosystems 7500 platform.

### (ii) HRV RNA infectivity studies.

H1 HeLa cells (CRL-1958) were used to assess the presence or absence of viral infectivity as visualized by CPE. Cells were propagated in six-well plates using DMEM supplemented with 10% fetal bovine serum and incubated at 35°C in 5% CO_2_ for 24 h. RNA transfection was performed using 10 µl of RNA, cDNA, or SISPA product following the TransIT-mRNA transfection kit protocol (Mirus). After transfection, cells were incubated at 35°C for 72 h, and CPE was observed daily. Samples determined to be CPE negative were used in passage 2. Specifically, 500 µl of supernatant from passage 1 was used to infect fresh cells at 35°C for 1 h. After 1 h, 1.8 ml of DMEM (1×) with 10% FBS was added to each well, and the cells were incubated at 35°C for 72 h. CPE was observed daily. Samples deemed CPE negative were used in passage 3 following the same protocol as that used for passage 2. For all experiments, an HRV-16 genomic RNA was transfected in parallel as a positive control for RNA infectivity. For large-scale loss of RNA infectivity studies, the SOP was performed on 44 HRV-16 samples and 44 HRV-14 samples in a 96-well format. Briefly, 12.5 µl from each well was used to make eight tubes of 137.5 µl (pooling material from A1-A11, B1-B11, C1-C11, etc.). H1 HeLa cells were propagated in petri dishes using DMEM supplemented with 10% fetal bovine serum. Next, 137.5 µl of SISPA products was transfected into each petri dish using the TransIT-mRNA transfection kit (Mirus). Cells were incubated at 35°C for at least 72 h and monitored daily for the appearance of CPE. If no CPE observed, 3 ml of supernatant from each petri dish was inoculated into fresh HeLa cells. This was repeated for a total of three passages. For all experiments, an HRV-16 genomic RNA was transfected in parallel as a positive control for RNA infectivity.

To determine the limit of detection for infectious RNA, RNA was isolated from 10-fold dilutions of HRV-16 virion particles. RNA was transfected onto H1 HeLa cells using the TransIT-mRNA transfection kit (Mirus) and subjected to three rounds of blind passaging. CPE was evident in all cells infected with at least 0.1 PFU by the second passage. No CPE was observed in cells infected with less than 0.01 PFU (data not shown). The limit of infectivity for infectious RNA was reproducible over multiple replicates.

### (iii) HRV16 viral infectivity studies.

For studies of large-scale loss of viral infectivity, the SOP was performed on 44 HRV-16 samples and 44 HRV-14 samples in a 96-well format. The final eluted volume was 50 µl and was divided into four aliquots. To reduce the amount of CPE testing, samples in all rows were pooled prior to CPE testing. Briefly, 12.5 µl from each well was used to make eight tubes of 137.5 µl (pooling material from A1-A11, B1-B11, C1-C11, etc.). Target H1 HeLa cells were propagated in T75 flasks using DMEM supplemented with 10% fetal bovine serum and incubated at 35°C. For infectivity studies, the supernatant was removed, and the pooled 137.5 µl was mixed with 3 ml of infection medium (1× DMEM plus 1% FBS). This mix was used to infect T75 flasks for 1 h, gently rocking flasks every 20 min. After 1 h, the medium was replaced, and flasks were incubated at 35°C for 72 h. If no CPE was observed, 3 ml of supernatant from each flask was inoculated into fresh HeLa cells. This was repeated for a total of three passages. For all experiments, infectious HRV-16 viral particles served as a positive control for CPE.

To determine the limit of detection for infectious virus, 10-fold dilutions of HRV-16 were inoculated onto H1 HeLa cells and subjected to three rounds of blind passaging as described above. CPE was evident in all cells infected with at least 0.1 PFU by the second passage. No CPE was observed in cells infected with less than 0.01 PFU (data not shown). The limit of infectivity for infectious virus was reproducible over multiple replicates.

### Alphavirus methods (alphavirus stocks, SISPA reactions, and infectivity assays).

Alphavirus SISPA testing was done using either an infectious-clone-derived GirdwoodS.A. strain of Sindbis virus (SINV) (41) or an infectious-clone-derived vaccine strain (181/25) of CHIKV (42), which was generously provided by Terry Dermody, Vanderbilt University. These infectious clones were engineered to express the mKate fluorescent protein followed by the FMDV 2a protease in frame between the viral capsid and E3 gene as previously described (43). All virus stocks were generated by production of virus length genomic RNA using SP6 mMessage mMachine RNA transcription kits (Ambion), which was electroporated into BHK-21 cells as described previously (44). Titers of each viral stock were determined using our standard plaque assay protocol on Vero cells (17).

SISPA reactions were tested for the presence of infectious SINV or CHIKV on Vero cells. Briefly, 15 µl of the pooled SISPA library was mixed with 1 ml of complete MEM (MEM plus 10% FBS, l-glutamine [l-glut], and penicillin-streptomycin [pen-strep]), and the Vero cells were placed in T25 flasks for 1 h, with rocking every 15 min at 37°C and 5% CO_2_. At the end of the 1-h incubation, 4 ml of complete medium was added to the flask. A mock control flask received complete medium alone. The first-passage monolayers (P:1) were maintained for 72 h, with the monolayers assessed for cytopathic effect and red fluorescence daily. At 72 h, 20% of supernatant from the passage 1 flask was introduced onto a new monolayer (P:2) using the same 1-h infection protocol, and the flasks were maintained for 72 h, with the monolayers assessed for CPE and red fluorescence every 24 h. Next, 20% of the P:2 supernatant was then placed on a third monolayer (P:3) and monitored for an additional 72 h. To assess assay sensitivity, the CPE protocol was also performed using 1 ml of complete medium containing 100, 10, 1, or 0.1 PFU of SINV or CHIKV. The assay sensitivity routinely ranged from 0.1 to 1 PFU.

To test alphavirus RNA inactivation in SISPA reactions, 10^7^ BHK cells were electroporated with 15 µl of the pooled SISPA SINV or CHIKV libraries, and seeded into T75 flasks with 15 ml MEM supplemented with 10% FBS, l-glut, and pen-strep. Monolayers were maintained for 72 h at 37°C and assessed for CPE and red fluorescence every 24 h. At 72 h, 3 ml (20%) of supernatant was transferred to a new T75 flask of Vero cells (P:2), which were monitored for CPE every day for 72 h, at which point 20% of the supernatant from P:2 was placed on a third monolayer (P:3) of Vero cells, which was assessed for CPE for 72 h.

### MERS-CoV methods. (i) Virus and cells.

The Jordan MERS-CoV strain (GenBank accession no. KC776174.1, MERS-CoV-Hu/Jordan-N3/2012) was kindly provided by Kanta Subbarao (National Institutes of Health, Bethesda, MD), Gabriel Defang (Naval Medical Research Unit 3 [NAMRU-3], Cairo, Egypt), Michael Cooper (Armed Forces Health Surveillance Center [AFHSC]) and Emad Mohereb (NAMRU-3). All experiments with live MERS-CoV (Jordan) were performed under biosafety level 3 conditions at the University of Maryland School of Medicine. Vero E6 (monkey kidney epithelial cells, ATCC CRL-1586) for plaque assays were grown in minimal essential medium (Corning) supplemented with 10% (vol/vol) FBS (Sigma Aldrich), 1% l-glutamine (Gibco) and 1% penicillin-streptomycin (Gemini Bio-Products) at 37°C in a 5% CO_2_ atmosphere.

### (ii) MERS-CoV quantification and safety testing.

To determine the titers of live MERS-CoV, media from infected cells and processed RNA were placed on Vero E6 cells to determine the titer in PFU per milliliter as previously described (45). To quantify the infectivity of samples throughout the described RNA isolation protocol, RNA or cDNA intermediates or final purified products were used to infect Vero E6 cells in a six-well plate (1 × 10^6^ cells per well). The cells were passaged every 48 h for 3 blind passages for each well, and CPE was examined.
