# Supplementary material for: A Universal Next-Generation Sequencing Protocol To Generate Noninfectious Barcoded cDNA Libraries from High-Containment RNA Viruses
Source: mSystems. 2016 Jun 7;1(3):e00039-15. doi: 10.1128/mSystems.00039-15 (PMC5069770; doi:10.1128/mSystems.00039-15)
Supplement: Text S1 [file sys003162028s5.docx]

| 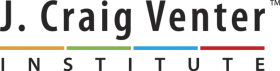 | | *Title:* | **Universal Standard Operating Procedure for Generation, Barcoding, and Amplification of cDNA from Genomic RNA of BSL-3/4 Viruses** | | | | |
| --- | --- | --- | --- | --- | --- | --- | --- |
| *Maintainer:* | Viral Programs | *Revision*: | DHS-SOP Parts A-Dv3.2 | *Revision Date:* |  | *Creation Date:* |  |

**Test Article/Sample Identification:**

Record sample identification(s) in the area below. Attach additional sheets to document samples as needed.

| (1) |
| --- |
| (2) |
| (3) |
| (4) |
| (5) |
| (6) |
| (7) |
| (8) |

**Purpose:**

The purpose of this document is

- To describe a Standard Operating Procedure (SOP) for the generation, barcoding, and amplification of cDNA from the genomic RNA of BSL-3/4 viruses
- To be used as a record of performance for the described procedure

**Important factors:**

- Follow appropriate agent specific biosafety guidelines: the pathogens under study are dangerous, so always work with infectious virus in the appropriate laboratory setting (e.g., BSL3) and in a certified biosafety cabinet (BSC) using the personal protective equipment and precautions required for the specific agent.
- Follow safety guidelines for reagents and solutions (Buffer RLT contains high concentration of guanidine salt).
- Keep samples and kit reagents on cold blocks or ice once thawed (cold blocks preferred).
  - Check for precipitates in reagents upon thawing, vortex to resuspend and flash spin if needed (sometimes heating is required, see specific manuals).
- Keep primer stocks free of contamination, aliquot for use by each individual laboratory worker.
- Document performance of each step as you go along, by checking off the appropriate boxes throughout this SOP.
- All equipment used in the performance of this procedure, including micropipettors, should be calibrated and maintained to be within normal operating specifications.

**Avoiding contamination:** Downstream techniques employ very sensitive PCR procedures so extreme care should be used to avoid contamination of the tubes, solutions etc. Additionally, once the procedure has been performed the tubes and contents are free of infectious agents and need to be handled appropriately to prevent contamination.

- Clean the bench area and replace any bench liners in preparation for non-infectious work. (Recommend 1-10% bleach, followed by 70% ETOH).
- If possible work in a dedicated “clean-biosafety cabinet” in which nucleic acid isolation is conducted, but the use of PCR amplification products, plasmids, and/or virus amplification is avoided. (The BSC should be cleaned using 1% bleach, followed by 70% ETOH).
- RNases are stable, ubiquitous, and on our skin so use gloves for everything (recommend nitrile).
- Use RNase/DNase-free tubes and handle them as little as possible.
- Label all RNA isolation and plastic ware containers so that all members of lab are aware to keep these items free of contaminating RNases or nucleic acids (e.g., “RNA only”). Recommend storing tubes and other plastic ware in the original containers that each user individually controls.
- Pour tubes from bag or beaker onto saran wrap, pickup carefully to avoid opening, close lid, label, and rack in advance of the procedures.
- Use dedicated tube openers to open microcentrifuge tubes, rather than hands.
- Use aerosol barrier tips for pipetting.
- Always setup an extraction negative control(s) to identify contamination (*this should be one of the last tubes in the rack or well on the plate*)

**Required Materials and Equipment:**

Record lot/batch numbers, expiration dates and equipment serial numbers for critical reagents and equipment as indicated below.

**Purification of Viral RNA:**

| Description of Material | Manufacturer | Catalog # | Lot/Batch Number | Expiration Date |
| --- | --- | --- | --- | --- |
| RNeasy Mini Kit | Qiagen | 74104 | N/A | N/A |
| - RNeasy Mini Spin Columns | Qiagen | N/A | N/A | N/A |
| - Collection Tubes (1.5 ml) | Qiagen | N/A | N/A | N/A |
| - Collection Tubes (2 ml) | Qiagen | N/A | N/A | N/A |
| - Buffer RLT | Qiagen | N/A |  |  |
| Buffer RW1 | Qiagen | N/A |  |  |
| Buffer RPE (concentrate)* | Qiagen | N/A |  |  |
| RNase-free Water | Qiagen | N/A |  |  |
| Molecular biology grade ethanol (100%) |  |  |  |  |
| RNase/DNase-free, aerosol resistant tips (1000 µl & 200 µl) | N/A | N/A | N/A | N/A |
| RNase-free 1.5 ml snap closure microcentrifuge tubes | N/A | N/A | N/A | N/A |
| Pipettes, 10 ml | N/A | N/A | N/A | N/A |
| 50 ml conical tubes | N/A | N/A | N/A | N/A |

* If using the RNeasy Mini Kit for the first time, add 4 volumes of ethanol (100%) as indicated on the RPE bottle to obtain a working solution.

| Description of Equipment | Serial Number |
| --- | --- |
| Biosafety cabinet |  |
| Micro-centrifuge (recommend refrigerated unit at 4°C if available) |  |
| Micropipettors (1000 µl & 200 µl) | N/A |
| Pipet Aid | N/A |

**SISPA cDNA Library Creation:**

| Description of Material | Manufacturer | Catalog # | Lot/Batch Number | Expiration Date |
| --- | --- | --- | --- | --- |
| Superscript III One-step RT-PCR System with Platinum Taq High Fidelity Polymerase | Life Technologies | 12574-035 |  |  |
| SuperScript III RT/ Platinum Taq High  Fidelity Enzyme Mix | Life Technologies | N/A |  |  |
| 2X Reaction Mix | Life Technologies | N/A |  |  |
| 5-mM Magnesium Sulfate | Life Technologies | N/A |  |  |
| Klenow Fragment (3’-5’ exonuclease) | New England Biolabs | M0212L |  |  |
| RNase H | New England Biolabs | M0297S |  |  |
| 10 µM stocks of N6-BC (random hexamer bar coded oligo) and corresponding BC primer (e.g., N6-BC1 and BC1) |  |  |  |  |
| RNase-free, DEPC-treated water |  |  |  |  |
| Molecular biology grade ethanol (100%) |  |  |  |  |
| RNase-free 0.2 ml PCR tubes or plates | N/A | N/A | N/A | N/A |
| RNase/DNase-free, aerosol resistant tips (1000 µl, 200 µl, 20 µl, & 2 µl) | N/A | N/A | N/A | N/A |
| Agarose gel reagents (optional) (agarose, TAE, & EtBr or pre-made gels, loading dye, ladder, etc.) | N/A | N/A | N/A | N/A |

| Description of Equipment | Serial Number |
| --- | --- |
| Thermocycler |  |
| Micropipettors (1000 µl, 200 µl, 20 µl, & 2 µl) | N/A |
| Pipet Aid | N/A |
| Agarose gel electrophoresis equipment (optional) | N/A |

**Inactivation of Residual Viral Genomic RNA:**

| Description of Material | Manufacturer | Catalog # | Lot/Batch Number | Expiration Date |
| --- | --- | --- | --- | --- |
| RNase Cocktail | Life Technologies | AM2286 |  |  |
| RNase/DNase-free, aerosol resistant tips (200 µl, 20 µl, & 2 µl) | N/A | N/A | N/A | N/A |

| Description of Equipment | Serial Number |
| --- | --- |
| Thermocycler |  |
| Micropipettors (200 µl, 20 µl, & 2 µl) | N/A |

**Purification of SISPA cDNA Library:**

| Description of Material | Manufacturer | Catalog # | Lot/Batch Number | Expiration Date |
| --- | --- | --- | --- | --- |
| QIAquick PCR Purification Kit | Qiagen | 28104 |  |  |
| QIAquick Spin Columns | N/A | N/A | N/A | N/A |
| Buffer PB |  |  |  |  |
| Buffer PE (concentrate)* |  |  |  |  |
| Buffer EB |  |  |  |  |
| Collection Tubes | N/A | N/A | N/A | N/A |
| RNase-free 1.5 ml snap closure microcentrifuge tubes | N/A | N/A | N/A | N/A |
| RNase-free 0.2 ml PCR tubes or plates | N/A | N/A | N/A | N/A |
| RNase/DNase-free, aerosol resistant tips (1000 µl, 200 µl, 20 µl, & 2 µl) | N/A | N/A | N/A | N/A |

* If using the QIAquick Kit for the first time, add ethanol (96–100%) to Buffer PE before use (see bottle label for volume).

| Description of Equipment | Serial Number |
| --- | --- |
| Micro-centrifuge (recommend refrigerated unit at 4°C if available) |  |
| Micropipettors (1000 µl, 200 µl, 20 µl, & 2 µl) | N/A |

**Heat Inactivation of SISPA cDNA Library:**

| Description of Equipment | Serial Number |
| --- | --- |
| Thermal Block |  |

**Left and Right Side SPRI Size Selection:**

| Description of Material | Manufacturer | Catalog # | Lot/Batch Number | Expiration Date |
| --- | --- | --- | --- | --- |
| SPRI select Reagent | Beckman Coulter | B23317 | N/A | N/A |
| Magnetic tube stand | N/A | N/A |  |  |
| Aglient Bioanalyzer | Aglient | N/A | N/A | N/A |

**METHODS:**

### RNA Purification Procedure

Isolation of RNA free of contaminants and RNases may be the most critical step for the amplification of overlapping dsDNA copies representing the viral genomes.

| Step | Description | Check if Performed |
| --- | --- | --- |
| 1 | Aliquot enough RLT for the total number of RNA purifications being done into a 50 ml conical tube (*e.g.*, 3.6 ml for 9 samples and 1 negative control). |  |
| 2 | Add 350 μl of RLT to each labeled individual snap-cap tube, place in biosafety cabinet. |  |
| 3 | Add 100 µl of virus-containing sample and pipette 5-times to mix. (*If you have less than 100 µl, adjust the volume up to a final of 100 µl using RNase-free ddH2O*) |  |
| 4 | Cap and mix well and incubate 10 minutes at room temperature. |  |
| 5 | Setup RNeasy Mini spin columns in 2 ml collection tubes (supplied) in a stable rack (make sure to label the column appropriately, *use alcohol resistant ink*). |  |
| 6 | Add 250 μl molecular biology grade ethanol (100%) to the diluted RNA, and mix well by pipetting 8-times, and transfer the sample (~700 μl) immediately to the spin columns placed in a 2 ml collection tube (setup in step 5). |  |
| 7 | Close the lid gently, and centrifuge for 15 s at 8000 x g. Discard the flow-through and return spin column to the same collection tube. (*Note: After centrifugation, carefully remove the RNeasy spin column from the collection tube so that the column does not contact the flow-through. Initial centrifugation (7-12) should be done at (20°C) and elution should be done at 4°C)* |  |
| 8 | Add 500 μl Buffer RPE (*ensure ethanol is added into the bottle before first use*) to the spin column. Close the lid, and centrifuge for 15 s at 8000 x g. Discard the flow-through. |  |
| 9 | Add 500 μl Buffer RPE to the spin column. Close the lid, and centrifuge for 2 min at 8000 x g. |  |
| 10 | Place the RNeasy spin column into a new 2 ml collection tube (supplied in kit), and discard the old collection tube with the flow-through. |  |
| 11 | Close the lid and centrifuge at max-speed (12,000-16,000 x g) for 1 min. |  |
| 12 | Carefully remove the column after centrifugation and place the RNeasy spin column into a new 1.5 ml collection tube (supplied in kit). |  |
| 13 | Add 30 μl RNase-free water to the center of the spin column membrane close the lid gently and let stand at room temp (18-25 C) for 1 minute. Place in pre-cooled centrifuge and spin for 1 min at 12,000-16,000 x g at 4°C to elute the RNA. *The caps of the tubes are likely to break off during this step, so be prepared to transfer the eluted RNA to a new well-labeled tube. If a cooled centrifuge is not available 18-25 C is acceptable for elution but the tubes containing the eluted RNA should be placed at 4 C immediately after centrifugation.* |  |
| 14 | Remove from centrifuge immediately and keep these tubes at 4°C or on ice while working in the lab and store the RNA at -80°C for future use. (*The RNA concentration is usually so low that the spectrophotometer is not useful for determining the concentration).*  Potential stopping point. If work is stopped and the RNA is stored, record the storage location (_____ ) and temperature (_____°C) |  |

**SISPA cDNA Library Creation**

In this step you will convert RNA from the sample into cDNA using barcoded (i.e. indexed) primers, then create dsDNA and amplify it using PCR.

| Step | Description | Check if Performed |
| --- | --- | --- |
| 1 | Isolate RNA as described above. |  |
| 2 | Turn on temperature cycler and hold at 50 *°C.* |  |
| 3 | Prepare Master reaction mixture on ice. Add H_2_O first and enzyme last.  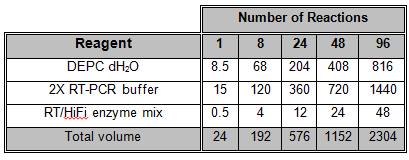  Volumes indicated as µl |  |
| 4 | Mix the contents of the master mix by pipetting. |  |
| 5 | Add 23.5 µl of Master Mix to tubes/wells at 4^o^C (cold block). |  |
| 6 | Add 1 µl of N6-BC primers; each tube/well will have a different primer (e.g., N6-BC1) |  |
| 7 | Add 5 µl RNA or dH_2_O (negative control) from a sample to each tube/well. (*Note sample identifiers and primer combinations need to be recorded because the specific bar code primer used relates to a specific sample*). |  |
| 8 | Transfer the reaction vessels to the pre-heated PCR machine (50°C) to perform the reverse transcription reaction. Cycle the temperatures as listed below:  50˚C, 5 minutes 🡪 4˚C, 5 minutes 🡪 25˚C, 15 minutes 🡪 50˚C, 30 minutes 🡪 55˚C, 10 minutes 🡪 70˚C, 15 minutes 🡪 4˚C, 10 minutes-1h (set machine to infinite). |  |
| 9 | Prepare Klenow, RNase H mixture (*this can be done during step 8*)  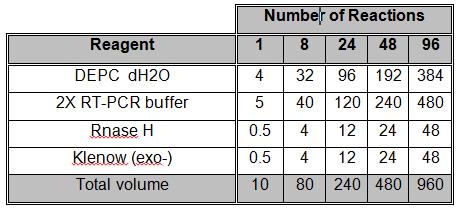  Volumes indicated as µl |  |
| 10 | Pipette the Klenow/RNase H mixture 3 times and add 9.5 µl to each RT reaction tube/well at 4 ˚C. |  |
| 11 | Place the reactions in temperature cycler at 37 °C and use following cycling parameters:  37˚C for 60 minutes 🡪 80˚C, 10 minutes 🡪 4 ˚C for 10 minutes to 1 hour |  |
| 12 | Keep samples at 4 ˚C and add **2 µl** of specific BC primer at 10 µM (e.g., BC-1) to each reaction/tube or well on ice (*the specific bar code primer used needs to correspond with the N6-BC primer used for the reverse transcription reaction).* |  |
| 13 | Transfer the reactions to a temperature cycler preset to 80˚C then use the following temperature cycling parameters:  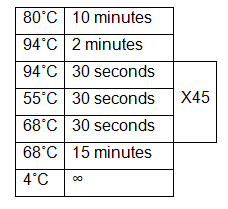 |  |
| 14 | Proceed to viral genome inactivation or store at -20 to -80 ˚C in freezer.  Potential stopping point. If work is stopped and the DNA is stored, record the storage location (_____ ) and temperature (_____°C) |  |

**Inactivation of Residual Viral Genomic RNA**

| Step | Description | Check if Performed |
| --- | --- | --- |
| 1 | Aliquot enough RNase Cocktail for all SISPA reactions. 2 µl of RNase Cocktail are required per tube/well  Volume of RNase Cocktail aliquoted: ___________ µl |  |
| 2 | Add 2 µl of RNase Cocktail to each reaction tube/well. |  |
| 3 | Incubate at 37 ˚C for 30 minutes, then 4 ˚C for 10 minutes and proceed to purification immediately or freeze at -20 ˚C.  Potential stopping point. If work is stopped and the DNA is stored, record the storage location (_____ ) and temperature (_____°C) |  |

**Purification of SISPA cDNA Library**

- At this point the material should be free of infectious virions and the RNA genome. Be sure to use “clean” (decontaminated) tubes, rotors, equipment etc. for these steps to avoid contaminating the material to be removed from the high containment laboratory.
- Before starting read through the QIAGEN QIAquick PCR Purification Kit Handbook and be sure all buffers are at the appropriate working concentrations.

| Step | Description | Check if Performed |
| --- | --- | --- |
| 1 | Aliquot enough buffer PB to purify all the SISPA reactions. 200 µl of buffer PB are required per tube/well. (*PB should be 5 volumes of SISPA*).  Volume of buffer PB aliquoted: ____________ µl |  |
| 2 | Add 200 µl of buffer PB to tubes/wells. |  |
| 3 | Add 35 µl of each SISPA reaction to the tubes/wells containing buffer PB. |  |
| 4 | Place a QIAquick spin column in a 2 ml collection tube (provided). |  |
| 5 | Apply the PB/sample mixture to the column and centrifuge for 1 min at  16,000 x g. |  |
| 6 | Discard flow-through and place the column back into the same 2 ml tube. |  |
| 7 | Add 0.75 ml buffer PE into the column, let it stand for 2 min, and centrifuge for 1 min at 16,000 x g |  |
| 8 | Discard flow-through and place the column back in the same tube. Centrifuge the column for an additional 1 min at 16,000 x g. |  |
| 9 | Make sure there is no liquid on the side or bottom of the column and place it in a clean 1.5 ml microcentrifuge tube. |  |
| 10 | Add 40 µl buffer EB to the center of the column and ensure it completely covers the membrane, let the column stand for 1 min, and centrifuge for 1 min at 16,000 x g. *Note: the lids of snap cap tubes often break off during this step, so you can use a screw cap centrifuge tube, or be sure to have well labeled tube that you can transfer the RNA into after elution from the column.* |  |
| 11 | Run 5 µl on a 1.5% agarose gel with known standards (e.g., 1 Kb+ ladder (Life Tech) to visualize size range of DNA products. |  |
| 12 | Use for subsequent procedures or store at  -20 to -80 °C. ( BSL3 lab)  Record the storage location (_____ ) and temperature (_____°C)  When ready to transfer the tube from BSL3 lab proceed to Heat Inactivation . |  |

**Heat Inactivation of SISPA cDNA Library**

- This step is performed immediately prior to transfer to BSL2 space to ensure no cross contamination of the final product.

| Step | Description | Check if Performed |
| --- | --- | --- |
| 1 | Turn on the Thermal block and set the Temperature at 72°C. |  |
| 2 | Transfer the 1.5ml micro centrifuge tube containing approx. 35ul of purified SISPA cDNA library to the preheated Thermal block at 72°C for 30 minutes.  Note: After Heat Inactivation - DO NOT OPEN THE TUBE. |  |
| 3 | Transfer the tube from BSL-3 lab following institutional procedures for surface decontamination (e.g. dunk tank) and store at -20 to -80 °C.  Record the storage location (_____ ) and temperature (_____°C) |  |

**Sample Pooling**

This step is performed to combine all individually barcoded SISPA products prior to size selection and the addition of Illumina Adaptors suitable for Illumina MiSeq, HiSeq, and/or NextSeq. Following quantification, an average concentration after completion of the SOP is approximately 20-40 nanograms per microliter. We recommend pooling material from at least 10 libraries but have pooled up to 200 libraries for a single MiSeq run. NOTE: when pooling, ensure all barcodes are different since identical barcodes will not be able to be demultiplexed following sequencing and during analysis.

| Step | Description | Check if Performed |
| --- | --- | --- |
| 1 | Based on the gel run in step 11 from the section entitled, “Purification of SISPA cDNA Library” you will know if the reaction was successful. Evaluate product from each SISPA sample |  |
| 2 | If a strong signal is detect, combine all barcoded SISPA products into a single RNase/DNAse free LoBind tube. NOTE: All barcodes from individual samples MUST be unique. |  |
| 3 | Proceed to the next step, “Size selection of pooled SISPA libraries” |  |

**Size Selection of Pooled SISPA Libraries**

SPRI select is a SPRI-based chemistry that speeds and simplifies nucleic acid size selection for fragment library preparation for Next-Generation sequencing. In this process, size selection is required to produce a uniform distribution of fragments ranging from 200-1000 basepairs in length. An example of size-selected, pooled SISPA products is displayed below. “Left side selection” refers to the removal of DNA fragments lower than 200 base pairs. “Right side selection refers to removal of DNA fragments greater than 1000 base pairs. A Bioanalyzer image following size selection is displayed below.

**
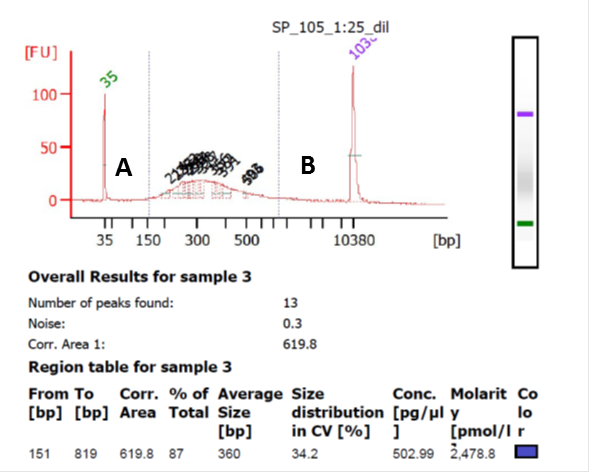
**

**Left image:** Example of a Bioanalyzer image of pooled SISPA products after completing the SOP followed by size selection (protocol for size selection is below). In the figure “A” indicates the removal of products following left side selection and “B” indicates the removal of DNA following right side selection.

**Left Side Size Selection**

| Step | Description | Check if Performed |
| --- | --- | --- |
| 1 | Thoroughly shake the SPRIselect bottle to resuspend the SPRI beads. Add the required volume of SPRIselect.  Volume of sample * 0.8x = Volume of SPRIselect  Example- 50ul of sample *0.8x = 40ul of SPRIselect. |  |
| 2 | Vortex for 1 minute at an appropriate speed (Note – Insufficient mixing of sample and SPRIselect will lead to inconsistent size selection results). Incubate at RT for 5 minutes. |  |
| 3 | Place the reaction vessel on an appropriate magnetic stand for 2-5 minutes and allow the SPRI beads to settle to the magnet. Settle times will vary, a higher initial sample volume or weak magnet will require a longer settle time. |  |
| 4 | Remove and discard the supernatant. (Note: Care should be taken not to aspirate more than trace amount of beads during this step, as the desired library is associated with the beads. Significant bead loss will result in reduced yield). |  |
| 5 | With the reaction vessel still on the magnet, add 180ul of freshly prepared 85% ethanol and incubate at RT for 30 seconds while rotating the tube. |  |
| 6 | Remove and discard the ethanol. Pulse spin the tube then place it back on the magnet. Use a P20 pipet with a fine tip to remove excess ethanol wash. |  |
| 7 | With the tube still on the magnet, air dry for up to 10 minutes. *Note: if beads appear dry before 10 minutes then time can be reduced.* |  |
| 8 | Remove the tube from the magnet and resuspend the beads in 50ul of Molecular biology grade water. Vortex and spin down tube. |  |
| 9 | Incubate at room temperature for 2 minutes. |  |
| 10 | Place the tube on the magnet for 2 minutes or until the supernatant has cleared. |  |
| 11 | Transfer supernatant to a new labeled LoBind tube and save 1ul for Agilent in a separate labeled LoBind tube (“Left side selection”). |  |

**Right Side Size Selection**

| Step | Description | Check if Performed |
| --- | --- | --- |
| 1 | Thoroughly shake the SPRIselect bottle to resuspend the SPRI beads. Add the required volume of SPRIselect.  Volume of sample * 0.65x = Volume of SPRIselect  Example- 50ul of sample *0.65x = 32.5ul of SPRIselect. |  |
| 2 | Vortex for 1 minute at an appropriate speed (Note – Insufficient mixing of sample and SPRIselect will lead to inconsistent size selection results). Incubate at RT for 2-5 minutes. |  |
| 3 | Place the reaction vessel on an appropriate magnetic stand for 5 minutes and allow the SPRI beads to settle to the magnet. Settle times will vary, a higher initial sample volume or weak magnet will require a longer settle time. |  |
| 4 | Transfer the clear supernatant, which contains the Right Side Size Selected sample to a new tube. The tube with remaining beads can be discarded. |  |
| 5 | Add the required volume of SPRIselect, using the calculation below, to the supernatant from the step 4 above. This will bind the fragments in the supernatant to the new SPRI beads.    Example- Sample Volume ul * 1.8x- the initial ratio = Volume of SPRIselect  **50ul + 32.5ul * 1.8-0.65 = 94.875ul** |  |
| 6 | Vortex for 1 minute at an appropriate speed (Note – Insufficient mixing of sample and SPRIselect will lead to inconsistent size selection results). Incubate at RT for 5 minutes. |  |
| 7 | Place the reaction vessel on an appropriate magnetic stand for 5 minutes and allow the SPRI beads to settle to the magnet. Settle times will vary, a higher initial sample volume or weak magnet will require a longer settle time. |  |
| 8 | Remove and discard the supernatant. (Note: Care should be taken not to aspirate more than trace amount of beads during this step, as the desired library is associated with the beads. Significant bead loss will result in reduced yield). |  |
| 9 | With the reaction vessel still on the magnet, add 180ul of freshly prepared 85% ethanol and incubate at RT for 30 seconds while rotating the tube. |  |
| 10 | Remove and discard the ethanol. Pulse spin the tube then place it back on the magnet. Use a P20 pipet with a fine tip to remove excess ethanol wash. |  |
| 11 | With the tube still on the magnet, air dry for up to 10 minutes. *Note: if beads appear dry before 10 minutes then time can be reduced.* |  |
| 12 | Remove the tube from the magnet and resuspend the beads in 50ul of Molecular biology grade water. Vortex and spin down tube. |  |
| 13 | Incubate at room temperature for 2 minutes. |  |
| 14 | Place the tube on the magnet for 2 minutes or until the supernatant has cleared. |  |
| 15 | Transfer supernatant to a new labeled LoBind tube. |  |
| 16 | Run 1ul of sample from Left Side Size Selection and 1ul from the final SISPA pool on Agilent to check for right size selection. |  |

**Illumina Adapter Ligation**

Adapter ligation is required prior to Illumina sequencing. The methods for adapter ligation are commercially available and catalogue numbers are indicated. Since sample barcodes are incorporated into the PCR products during the SOP and all samples are pooled prior to Illumina adaptor ligation, only a single Illumina adaptor set is added onto the final pool. Illumina adapters were ligated to pooled, size selected products using the New England Biolabs (NEB) end prep and ligation modules (E7546S, E7595S) and Bio Scientific barcoded adapters (514113). Libraries were purified and side selected using Agencourt AMPure XP (BECKMAN-COULTER A63882). Finally, Libraries were visualized on the Bioanalyzer using High sensitivity DNA chip (Agilent 5067-4626) and quantified using Kapa Illumina Library Quantification Kit (KK4835). While the SOP has been validated on Illumina MiSeq, HiSeq, and NextSeq, the Illumina MiSeq platform provides optimal results for viral genomes. For the MiSeq, we typically load 9pM of a final library pool on a MiSEq 2*300 cycle reagent cartridge for sequencing (MS-102-3003).
