# Supplementary material for: A Universal Next-Generation Sequencing Protocol To Generate Noninfectious Barcoded cDNA Libraries from High-Containment RNA Viruses
Source: mSystems. 2016 Jun 7;1(3):e00039-15. doi: 10.1128/mSystems.00039-15 (PMC5069770; doi:10.1128/mSystems.00039-15)
Supplement: Table S3 [file sys003162028st4.docx]

Supplemental Table 3. Heat treatment abolishes ssRNA+ viral infectivity. An additional heat inactivation step was included to further reduce the risk of residual infectious material leaving BSL-3/4 containment. Heat treatment at 72°C for 30 minutes completely abolishes infectious HRV-16, WNV, and all seven FMDV serotypes.

| Virus Family (Strain or Serotype) | Input Titer | Heat at 72° for 30 minutes | Ambient temperature for 30 minutes |
| --- | --- | --- | --- |
|  | PFU/ml^1^  TCID50/ml^2^ | Viral infectivity tests  Number CPE positive/Number tested | |
| Picornavirus (HRV-16) (A) | 1.00E+06^1^ | 0/4 | 4/4 |
| Picornavirus (HRV-16) (A) | 1.00E+04^1^ | 0/4 | 4/4 |
| Flavivirus (WNV) (A) | 1.00E+05^1^ | 0/4 | 4/4 |
| Picornavirus (FMDV SAT1) | 3.16E+04^2^ | 0/3 | 3/3 |
| Picornavirus (FMDV SAT2) | 5.62E+04^2^ | 0/3 | 3/3 |
| Picornavirus (FMDV SAT3) | 1.78E+05^2^ | 0/3 | 3/3 |
| Picornavirus (FMDV C3 Resende) | 1.00E+05^2^ | 0/3 | 3/3 |
| Picornavirus (FMDV Asia 1) | 1.78E+05^2^ | 0/3 | 3/3 |
| Picornavirus (FMDV A5 Westerward) | 5.62E+05^2^ | 0/3 | 3/3 |
| Picornavirus (FMDV O1 Caseros) | 3.16E+06^2^ | 0/3 | 3/3 |

1. For these samples, viral infectivity was also abolished following heat at 72°C for either 5 minutes or 60 minutes
